# Lipidome Profiling in Childhood Obesity Compared to Adults: A Pilot Study

**DOI:** 10.3390/nu15153341

**Published:** 2023-07-27

**Authors:** Andrea Soria-Gondek, Pablo Fernández-García, Lorena González, Marjorie Reyes-Farias, Marta Murillo, Aina Valls, Nativitat Real, Silvia Pellitero, Jordi Tarascó, Benjamin Jenkins, María Galán, Francesc Villarroya, Albert Koulman, Patricia Corrales, Antonio Vidal-Puig, Rubén Cereijo, David Sánchez-Infantes

**Affiliations:** 1 Pediatric Surgery Department, Hospital Universitari Germans Trias i Pujol, 08916 Badalona, Spain; andreasoriagondek@gmail.com; 2 Department of Health Sciences, Campus Alcorcón, University Rey Juan Carlos (URJC), 28922 Madrid, Spain; pablo.fernandezga@urjc.es (P.F.-G.); mgalana@urjc.es (M.G.); patricia.corrales@urjc.es (P.C.); 3 Fundació Institut Germans Trias i Pujol, 08916 Barcelona, Spain; lorenagl.lorena@gmail.com (L.G.); marjorie.reyesfarias@ub.edu (M.R.-F.); 4 Department of Biochemistry and Physiology, School of Pharmacy and Food Sciences, Institut de Biomedicina de la Universitat de Barcelona (IBUB), Universitat de Barcelona, 08028 Barcelona, Spain; 5 Pediatric Endocrinology Unit, Pediatric Department, Hospital Universitari Germans Trias i Pujol, 08916 Badalona, Spain; mmurillo.germanstrias@gencat.cat (M.M.); ainavalls@hotmail.com (A.V.); 6 Pediatric Nurse, Hospital Universitari Germans Trias i Pujol, 08916 Badalona, Spain; nereal.germanstrias@gencat.cat; 7 Endocrinology Department, Hospital Universitari Germans Trias i Pujol, 08916 Badalona, Spain; spellitero.germanstrias@gencat.cat; 8 General Surgery Department, Hospital Universitari Germans Trias i Pujol, 08916 Badalona, Spain; jtarasco@me.com; 9 NIHR BRC Core Metabolomics and Lipidomics Laboratory, Metabolic Research Laboratories, Institute of Metabolic Science, University of Cambridge, Cambridge CB2 1GG, UK; bjj25@medschl.cam.ac.uk (B.J.); ak675@medschl.cam.ac.uk (A.K.); 10 Biochemistry and Molecular Biomedicine Department, Institut de Biomedicina de la Universitat de Barcelona (IBUB), 08028 Barcelona, Spain; fvillarroya@ub.edu; 11 Centro de Investigación Biomédica en Red de Fisiopatología de la Obesidad y Nutrición (CIBERobn), 28029 Madrid, Spain; 12 Institute of Metabolic Science, Addenbrooke’s Hospital, University of Cambridge, Cambridge CB2 1GG, UK; ajv22@medschl.cam.ac.uk; 13 Institut de Recerca Hospital de la Santa Creu i Sant Pau, 08041 Barcelona, Spain

**Keywords:** lipidomics, childhood obesity, bariatric surgery

## Abstract

The objective is to assess the circulating lipidome of children with obesity before and after lifestyle intervention and to compare the data to the circulating lipidome of adults with obesity before and after bariatric surgery. Ten pediatric (PE) and thirty adult (AD) patients with obesity were prospectively recruited at a referral single center. The PE cohort received lifestyle recommendations. The AD cohort underwent bariatric surgery. Clinical parameters and lipidome were analyzed in serum before and after six months of metabolic intervention. The abundance of phosphatidylinositols in the PE cohort and phosphatidylcholines in the AD significantly increased, while O-phosphatidylserines in the PE cohort and diacyl/triacylglycerols in the AD decreased. Fifteen lipid species were coincident in both groups after lifestyle intervention and bariatric surgery. Five species of phosphatidylinositols, sphingomyelins, and cholesteryl esters were upregulated. Eight species of diacylglycerols, glycerophosphoglycerols, glycerophosphoethanolamines, and phosphatidylcholines were downregulated. Most matching species were regulated in the same direction except for two phosphatidylinositols: PI(O-36:2) and PI(O-34:0). A specific set of lipid species regulated after bariatric surgery in adult individuals was also modulated in children undergoing lifestyle intervention, suggesting they may constitute a core circulating lipid profile signature indicative of early development of obesity and improvement after clinical interventions regardless of individual age.

## 1. Introduction

Nowadays, 2.1 billion people worldwide are affected by obesity and/or overweight [1]. In the context of this pandemic, global prevalence of obesity/overweight in children has increased tenfold in the past 40 years [2]. The decreasing age for the onset of obesity and obesity-related metabolic abnormalities such as cardiovascular disease (CVD), type 2 diabetes mellitus (T2DM), and premature death [3,4] has alarming negative public health implications [5,6], thus compelling the need for scientific research on childhood obesity.

Lipidomics is a relatively recent research technology that involves identifying and quantifying the thousands of cellular lipid molecular species and their interaction with other metabolites. Lipids represent approximately 50% of the mass of most animal cell membranes [7] and play a vital role as substrates for energy production and efficient storage [8], regulation and signaling [9,10], and membrane biophysical properties [11]. The lipidomic profile of adipose tissue has been shown to be depot-specific. In homeostatic conditions, the cell membranes and lipid droplets of adipocytes maintain remarkable control of their composition through specific mechanisms despite being exposed to high-fat diet, cold, or exercise [12,13]. Conversely, obesity involves quantitative and qualitative changes in human lipidic composition. When overloaded, adipocytes lose the ability to properly conduct lipid-storing functions [14], leading to ectopic lipid accumulation in the skeletal muscle, liver, pancreatic beta cells, or vascular walls [15]. This lipotoxicity leads to alterations in signaling pathways and allostatic responses to maintain energy homeostasis responsible for insulin resistance and other obesity-related complications [16,17]. Furthermore, obesity induces characteristic changes in serum lipid composition related to the pathogenesis of metabolic alterations [18,19,20,21]. Most of the available literature focuses on the metabolic alterations of adult obesity, but pathogenic mechanisms of childhood obesity warrant further investigation.

Here, we aimed to assess the circulating lipidome of children with obesity before and after lifestyle intervention, generating for the first time a lipidomic signature profile on the changes caused by that. Lipidomics of adults with obesity after bariatric surgery, which has been extensively studied before, was added as a reference baseline to assess possible differences and similarities between both approaches. We posited that coincident changes in lipid species of children and adults with obesity before and after their respective interventions should be further studied as potential molecules involved in the early development of obesity and lipotoxicity.

## 2. Methods

### 2.1. Study Cohorts

#### 2.1.1. Pediatric (PE) Cohort

Pediatric obesity was defined as BMI percentile ≥95th% for age- and sex-matching reference populations [22]. To enter the study, the pediatric patients and their families had to give their consent for the entire study protocol. Ten pediatric patients with obesity (age range 10–18) were recruited and received diet and lifestyle recommendations, the first-line treatment of pediatric obesity according to current national guidelines [23]. We must take into account that in order to participate in this study, the pediatric patients and their families had to accept two conditions exclusively linked to this study. Firstly, they had to agree to undergo two blood tests that had not been performed outside the study context. Secondly, they had to commit to coming to the clinic in person every month. This requirement primarily affected families with a low socioeconomic status, as they could not afford to come to the clinic on a monthly basis due to personal, family, and work-related reasons. These two requirements explain why out of the 25 potential patients, only 10 could be recruited. Nine out of ten pediatric patients were over 11 years old, of which eight were over 13 years old. Of the sample, 90% of patients had reached pubertal status.

The PE cohort received a protocolled diet, exercise, and lifestyle recommendations (Supplementary File S1) and underwent monthly follow-up visits to monitor the changes in lifestyle habits and to work on positive reinforcement. During the follow-up visits, a pediatric endocrinologist and a pediatric nutritionist conducted a standardized interview about self-reported dietary intake and exercise (Supplementary File S2). Clinical data (demographics and anthropometrics) before (0 M) and after (6 M) the lifestyle intervention were retrieved from routinely conducted clinical measurement databases. Characteristics of the PE cohort are indicated in Table 1.

#### 2.1.2. The Adult (AD) Cohort

Thirty adult patients with obesity (age range 18–65) underwent bariatric metabolic surgery, either sleeve gastrectomy or Roux-en-Y gastric bypass. Clinical data (demographics and anthropometrics) before (0 M) and after six months (6 M) of the surgical intervention were retrieved from routinely conducted follow-up clinical measurements (Supplementary File S2). Characteristics of the AD cohort are indicated in Table 1 as well.

### 2.2. Ethical Statement

The Institutional Ethics Committee (Germans Trias i Pujol CEIC), in accordance with the Declaration of Helsinki, approved the study (code PI17/01455 and PI20/00807). All participants gave their written informed consent before collecting clinical data and samples.

### 2.3. Serum Measurements

Serum samples were drawn after a 12 h fasting period at 0 M and 6 M from PE and AD cohorts to run a blood chemistry panel and a circulating lipid profile. The 0 M–6 M PE and 6 M AD samples were taken at the clinic. The 0 M AD samples were taken on the date of surgery. Insulin, glucose, HOMA-IR (homeostatic model assessment insulin resistance), glycated hemoglobin (HbA1c), urea, creatinine, AST (aspartate aminotransferase), ALT (alanine aminotransferase), GGT (gamma-glutamyl transpeptidase), TG (triglycerides), cholesterol, HDL-c (high-density lipoprotein-cholesterol), LDL-c (low-density lipoprotein-cholesterol), and total protein were measured in the core clinical laboratory at the hospital Germans Trias i Pujol, Badalona, Spain. The fatty liver index (FLI) was calculated with the following formula (*WC*: waist circumference):

$$FLI=\frac{e^{0.953\cdot \ln\left(TG\right)+0.139\cdot BMI+0.718\cdot \ln\left(GGT\right)+0.053\left(WC\right)-15.754}}{1+e^{0.953\cdot \ln\left(TG\right)+0.139\cdot BMI+0.718\cdot \ln\left(GGT\right)+0.053\left(WC\right)-15.754}}\cdot 100$$

Analyses of the lipidomes in these samples were performed at the same time in all serum samples from PE and AD cohorts as follows.

### 2.4. Lipid Extraction

The phospholipid, triglyceride, and sterol fractions were isolated together using a recently reported method. Briefly, dried blood spot samples were placed along with blank and quality control samples (QCs) in the wells of a glass-coated 2.4 mL/well ninety-six-well plate (96 w plate; Plate+™, Esslab, Hadleigh, UK). Water (100 μL, MilliQ) was added to each of the wells, followed by methanol (250 μL, HPLC grade, spiked with 0.6 μM 1,2 di-O-octadecyl-sn-glycero-3-phosphocholine, 1.2 μM 1,2-di-O-phytanyl-sn-glycero-3-phosphoethanolamine, 0.6 µM C8-ceramide, 0·6 µM *n*-heptadecanoyl-d-erythro-sphingosylphosporylcholine, 6.2 µM undecanoic acid, 0.6 μM trilaurin), followed by tert-butyl methyl ether (TMBE, 500 μL). The plates were then sealed (aluminium microplate sealing tape), agitated (10 min, 600 rpm), and centrifuged for 10 min at 3200 g. A 96-head micro-dispenser was used to transfer 200 μL of the organic solution to a glass-coated 240 µL 96 w plate (Plate+™, Esslab, Hadleigh, UK). The plate was transferred to a Genevac EZ-2 evaporator (Genevac Ltd., Ipswich, UK) and dried. The samples were reconstituted (TBME, 25 μL and MS-mix [7.5 mM ammonium acetate in IPA:CH3OH (2:1)], 90 μL) using a Hydra Matrix, after which the plate was sealed and stored at −20 °C.

### 2.5. Mass Spectrometry

All samples were infused into an Exactive Orbitrap (Thermo, Hemel Hampstead, UK), using a Triversa Nanomate (Advion, Ithaca, NY, USA) and a mass resolution of 100,000 [24]. Samples were ionized at 1.2 kV. The Exactive started acquiring data 20 s after sample aspiration began. The phospholipid and triglyceride signals obtained were relative abundance (‘semi-quantitative’), with the signal intensity of each lipid expressed relative to the total lipid signal intensity, for each individual, per cent (%). Raw high-resolution mass-spectrometry data were processed using XCMS (www.bioconductor.org) and Peakpicker v 2.0 (an in-house R script). Quality control was based on a previously published approach [25]. Lipid signals that were not reliably detectable or did not show a linear response were removed. Lipid signals that were present in fewer than 80% of all QC samples or that had a poor correlation with concentration within the dilution range of QC samples (Pearson correlation *r* < 0.95) were removed. The coefficient of variation (CV) for each lipid signal was then determined and all lipids with CVs of more than 25% were omitted.

## 3. Statistical Methods

### 3.1. Descriptive Analyses

Clinical data from PE and AD cohorts at 0 M and 6 M were analyzed. Data normality for each subset was assessed with the Shapiro-Wilk and Kolmogorov-Smirnoff tests. The presence of outliers was assessed for all variables, time points, age groups, and differences with the ROUT method. Paired Student’s *t* (for parametrically distributed variables) or Wilcoxon signed rank (non-parametrically distributed) tests were used for the comparison of paired data. A *p*-value < 0.05 was set as the statistical significance threshold. For categorical variables, frequencies (total and percentage) and exact 95% confidence interval were calculated, and statistical significance was assessed with the Chi-square test. For continuous variables, mean and standard deviation (mean (sd)) were calculated. The correlation between age or sex and clinical variables was assessed using Pearson’s (parametric) or Spearman’s (nonparametric) linear regression.

### 3.2. Lipidomic Profile at 0 M and 6 M in the PE and AD Cohorts

Statistical analysis was performed using the statistical language “R” (R version 4.0.3 (10 October 2020), The R Foundation for Statistical Computing ©, Vienna, Austria) and the Bioconductor Project libraries for omics data analysis. All values were scaled by log transformation to normalize the great dispersion of lipid intensities. The selection of lipids with differential concentrations after different experimental conditions was based on a linear model adjusted by the empirical Bayes moderation of the variance [26]. To adjust for random effects associated with samples obtained from the same patient, an inter-subject correlation factor was calculated and included in the linear model [27]. In order to deal with the multiple testing issues derived from the fact that many tests (one per lipid) were performed simultaneously, *p*-values were adjusted to obtain strong control over the false discovery rate using the Benjamini and Hochberg method. The results of the production lipidomics were further adjusted by age and sex. Lipid names were converted to a more simplified nomenclature and grouped by superclass, main class and subclass according to data from the LipidLynxX database. Species with *p*-adjusted values <0.05 were considered and ordered by log2FC, in which positive values were upregulated at 6 M and negative were downregulated. Top tables with the lipids ranked from the most to the least differentially expressed, volcano plots depicting the relation between fold change and level of significance, and heatmaps depicting expression profiles of lipids were constructed for each comparison evaluated. Species from each assigned superclass, main class, and subclass were counted for each group (PE and AD, up- and downregulated) and plotted as stacked column graphs. Coincident lipidic species between PE and AD cohorts were also explored.

### 3.3. Lipidomics Pathway Assessment

Raw data from lipidomics were conveniently formatted and analyzed with BioPan (www.lipidmaps.org/biopan (accessed on 8 August 2022)) [28]. Species were processed or not for further analyses according to BioPan limitations (~170 processed species/~650). For the PE and AD cohorts, connection graphs were generated for activated and suppressed pathways. Statistical analyses were performed for paired data, establishing *p* < 0.05 as the threshold (BioPan’s Zscore > |1.645|). Data from the genes potentially involved were also retrieved.

### 3.4. Correlation between Clinical Variables and Differentially Regulated Lipids

To study the correlation between the main clinical variables and the differentially regulated lipids, the Spearman’s rho correlation index was calculated. Lipids from the top tables created in the lipidomic analysis that matched the following criteria were chosen for further analyses: (1) adjusted *p*-value < 0.01 and (2) absolute logarithmic fold change > 3. Twenty-nine lipids were selected from the comparison PE(6 M) vs. PE(0 M) and AD(6 M) vs. AD(0 M) (Supplementary File S3). The clinical variables included in the correlation analysis were height, weight, waist circumference, body mass index (BMI), insulin, glucose, HOMA-IR, glycated hemoglobin (HbA1c), urea, creatinine, ALT, GGT, bilirubin, TG, cholesterol, HDL-c, LDL-c, and total protein. A plot with the calculated correlation indexes between the differences at 6 M relative to 0 M in clinical variables and those in differentially expressed lipids from both groups was performed. Age and sex-adjusted multiple testing *p*-values and false detection rates (FDR) were calculated for each correlation and *p* < 0.05 was established as the statistic for significance assessment. To assess possible associations of lipidomic changes with BMI reductions in the PE cohort, patients were separated into two categories according to the following clinical criteria: (1) body weight reduction of >0.5 kg, (2) BMI reduction of 5%, and/or (3) decrease of a BMI percentile. Four out of the ten individuals in the PE cohort met these criteria and were thus considered responders to the lifestyle intervention in terms of BMI reduction. Age- and sex-adjusted multinomial logistic regression was conducted between differential levels of significantly regulated lipid species 6 M-0 M and successful BMI reduction (dichotomic), establishing the threshold of statistical significance at *p* < 0.05.

## 4. Results

### 4.1. PE Cohort

The resulting behavioral changes after lifestyle intervention in the PE cohort are summarized in Supplementary File S4. At 0 M, 7 patients had a BMI above reference 99th percentile, 2 above the 98th percentile, and 1 above the 97th percentile. At 6 M, 6 patients had a BMI above the 99th percentile, 2 above the 98th percentile, 1 above the 97th percentile, and 1 above the 92nd percentile. All abnormally upregulated clinical variables of interest, including body weight, WC, FLI, BMI, and HOMA-IR, trended to decrease between 0 M and 6 M after this intervention, albeit not statistically significantly except for LDL-c (114.9 vs. 105.4, *p* = 0.04) (Table 1). No age- or sex-dependent associations were observed with clinical variables in this PE cohort. Even though adiponectin and leptin levels were not statistically different at 6 M, we observed a significant decrease of TNF-α, MCP1, and IL-6 levels (Supplementary File S5).

**Figure 1:**
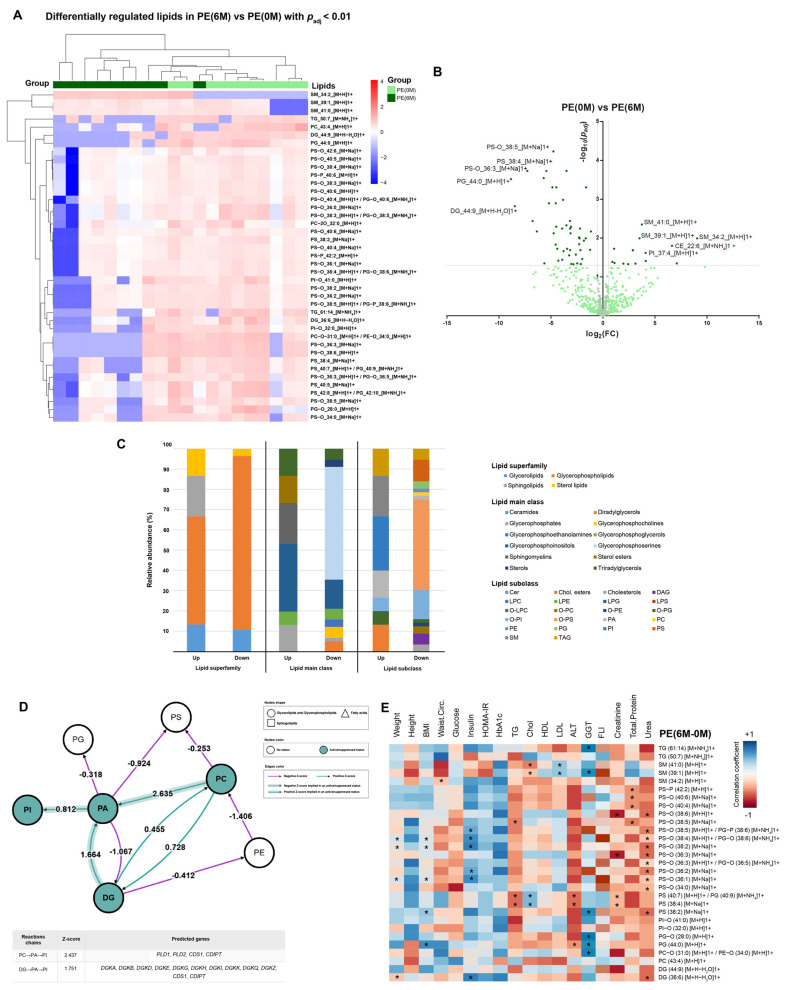
Overview of serum lipidomics in children with obesity after interventions aimed at weight loss. (**A**) A heatmap plot depicting significantly differentially regulated lipid species between 0 and 6 months of intervention and sample clustering in the pediatric (PE) cohort. (**B**) A volcano plot of statistically significant differentially regulated lipid species (6 M-0 M) in the PE cohort. Lipids are shown in a darker green tone for adjusted *p*-value < 0.01, and the absolute logarithmic fold change is >0.5 units. The most significant and regulated lipids are specifically labelled. (**C**) Relative abundance of upregulated and downregulated lipid species between 0 and 6 months in the PE cohort, classified according to top represented lipid superfamilies (left), main classes (center), and subclasses (right). (**D**) The pathway of phospatidylinositol biosynthesis activation 0 M-6 M from phosphatidic acid derived from diacylglycerols or phosphatidylcholine in the PE cohort. Interpretation information for each node and arrow shape and color is included. A measure of the effect is indicated for each arrow (*Z*-score values). Statistical analyses for paired data were performed, establishing *p* < 0.05 as the significance threshold. Significantly regulated pathways are indicated with a shaded arrow. For each pathway analysis, a table depicting the specific statistically significant reaction steps affected in the pathway, their associated *Z*-score, and predicted regulated genes involved in each step is included. (**E**) A heatmap depicting correlation coefficient (*r*) values for linear regressions between the differences of selected clinical variables and those of top significantly regulated lipids in the PE cohort after metabolic intervention (Δ6 M-0 M). Blue cells indicate positive (*r* > 0) and red cells indicate negative associations (*r* < 0). *: Significant correlations (*p* < 0.05).

Furthermore, significant changes in serum lipid composition were observed after 6 M of intervention as shown in Figure 1A; 71 lipid species showed differential changes in serum concentrations (15 upregulated and 56 downregulated). Specifically, PI(37:4), SM(41:0), SM(39:1), SM(34:2), and CE(22:6) were the lipids of which the circulating levels increased the most, and PS(O-36:3), PS(38:4), PS(O-38:5), PG(44:0), and DG(44:9) were the lipids of which the circulating levels decreased the most (Figure 1B). Overall, the top-upregulated lipid superfamily/mainclass/subclass were, respectively, glycerophospholipids, glycerophosphoinositols, and phosphatidylinositols (PI), while the top-downregulated were glycerophospholipids, glycerophosphoserines, and O-phosphatidylserines (PS O) (Figure 1C). Of note, these changes in circulating lipids after 6 M were independent of the most noticeable BMI reductions also occurring in 40% of participants (Supplementary File S8).

No lipidomic pathways were significantly repressed after 6 M of intervention. However, phosphatidylinositol biosynthesis from phosphatidic acid was significantly activated according to the lipid class analysis (Figure 1D).

Correlation analyses revealed some associations between regulation of different lipid species and clinical parameters including weight, BMI, waist circumference, insulinemia, cholesterol, TG, LDL-c, renal function, and hepatic markers (Figure 1E, Table 2). At 0 M, waist circumference was negatively correlated with adiponectin levels, whereas insulin, HOMA-IR, and TG were positively correlated with TNF-α levels. At 6 M, TG positively correlated with TNF-α levels, whereas HDL was negatively correlated with IL-8 levels (Supplementary File S5). No significant correlations with FLI variation and the selected lipids were observed in the PE cohort, with only a negative association with PI(O-40:2) occurring in the AD cohort (Figure 1E, Supplementary File S6). However, abundant and mainly positive significant correlations were found between FLI and some detectable species of DAG in this cohort. Such correlations exhibit a differential pattern after 6 M of metabolic intervention compared to those in the 0 M baseline, favoring associations with longer-chain species in the former. Of note, fewer significant correlations between FLI and DAG were detected in the AD cohort, which in turn differed from those observed in the PE cohort (Supplementary File S7).

### 4.2. AD Cohort

The AD cohort showed a more favorable body composition and metabolic profile at 6 M after bariatric surgery, with significant improvements in weight, waist circumference, BMI, fasting glucose, glycated hemoglobin (HbA1c), urea, AST, ALT, GGT, TG, LDL-c, cholesterol, FLI, and HDL-c (Table 1). No significant differences in the degree of improvement were observed depending on the type of surgical intervention. Adiponectin and leptin levels returned to normal levels along with a significant increase in MCP1 (Supplementary File S5). Of note, weight, bilirubin, cholesterol, HDL-c, LDL-c, creatinine, delta LDL, and GGT were associated with sex, whereas weight, urea, creatinine, and delta HDL correlated with age, so these variables were taken into consideration for further analyses (Table 2).

**Figure 2:**
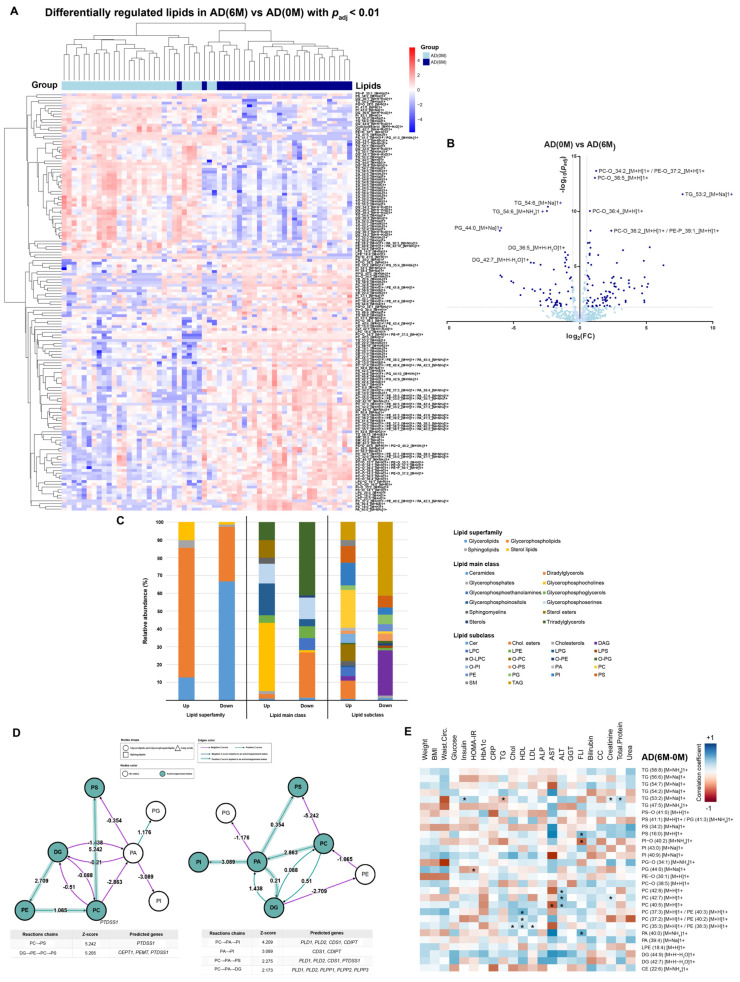
Overview of serum lipidomics in adult individuals with obesity after bariatric surgery. (**A**) A heatmap plot depicting significantly differentially regulated lipid species between 0 and 6 months of bariatric surgery and sample clustering in the adult (AD) cohort. (**B**) A volcano plot of statistically significant differentially regulated lipid species (6 M-0 M) in the AD cohort. Lipids are shown in a darker green tone for adjusted *p*-value < 0.01 and the absolute logarithmic fold change is >0.5 units. The most significant and regulated lipids are specifically labelled. (**C**) Relative abundance of upregulated and downregulated lipid species between 0 and 6 months of bariatric surgery in the AD cohort, classified according to top represented lipid superfamilies (left), main classes (center), and subclasses (right). (**D**) Pathway of phosphatidylserine biosynthesis activation (left) and of phosphatidylinositol and diacylglycerol biosynthesis repression 0 M-6 M (right) in the AD cohort. Interpretation information for each node and arrow shape and color is included. A measure of the effect is indicated for each arrow (*Z*-score values). Statistical analyses for paired data were performed, establishing *p* < 0.05 as the significance threshold. Significantly regulated pathways are indicated with a shaded arrow. For each pathway analysis, a table depicting the specific statistically significant reaction steps affected in the pathway, their associated *Z*-score, and predicted regulated genes involved in each step is included. (**E**) A heatmap depicting correlation coefficient (*r*) values for linear regressions between differences of selected clinical variables and those of top significantly regulated lipids in the AD cohort after bariatric surgery (Δ6 M-0 M). Blue cells indicate positive (*r* > 0) and red cells indicate negative associations (*r* < 0). *: Significant correlations (*p* < 0.05).

Significant changes in serum lipid composition of the AD cohort were also observed after 6 M of intervention (Figure 2A); 193 lipid species showed differential serum concentrations (118 upregulated and 75 downregulated). Specifically, PC(O-34:2)/PE(O-37:2), PC(O-36:5), PC(O-36:4), PC(O-36:2)/PE(P-39:1), and TG(53:2) were the lipids the circulating levels of which increased the most, while DG(36:5), TG(54:6), TG(54:6), DG(42:7), and PG(44:0) were the ones of which the circulating levels decreased the most (Figure 2B). The top-upregulated lipid superfamily/main class/subclass were glycerophospholipids, glycerophosphocholines, and phosphatidylcholines (PC) (Figure 2C), while the most downregulated were glycerolipids, di/triradylglycerols, and triacylglycerol/diacylglycerols.

Lipidomic pathway regulation analyses showed that phosphatidylserine biosynthesis became activated after the intervention, while phosphatidylinositol and DAG biosynthesis were repressed (Figure 2D). These results are in concordance with the lipid class analyses, in which DAG species abundance is reduced in favor of an increase of phosphatidylethanolamines and phosphatidylcholines. Due to the limitations of BioPan, no analyses could be conducted for mono/di/triglyceride pathways [28]. The predicted involved genes are also listed in Figure 2D.

Circulating levels of PC positively correlated with cholesterol, LDL-c, HDL-c, ALT, and AST, and negatively with total protein levels. On the other hand, 6 M-0 M changes in circulating levels of TG were positively associated with insulin, total protein, and urea, and negatively correlated with HbA1c (Figure 2E, Table 2). At 0 M, positive correlations were found between weight and MCP1 levels, BMI and leptin levels, waist circumference and TNF- α and MCP1 levels, and CRP and leptin and MCP1 levels. At 6 M, positive correlations were found between weight and leptin, CRP and leptin and MCP1, TG and MCP1, and HDL and adiponectin. On the other hand, BMI and waist circumference were negatively correlated with adiponectin levels (Supplementary File S5).

### 4.3. Lipidomic Profile at 0 M and 6 M in PE and AD Cohorts

**Figure 3:**
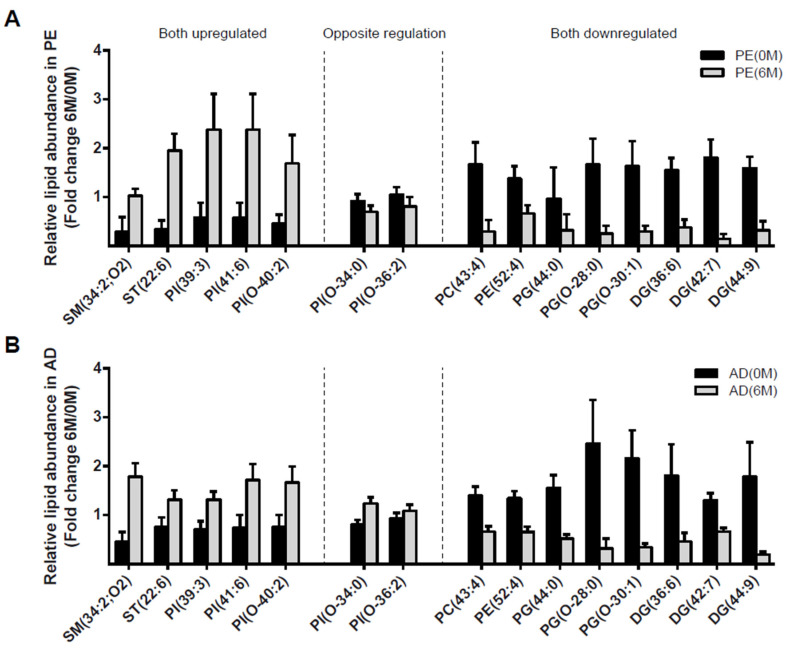
Coincident regulated lipid species in the PE and AD cohorts. Relative abundance (fold change 6 M-0 M) at 0 M (grey bars) and 6 M (black bars) of the 15 upregulated, oppositely regulated, or downregulated lipid species in both cohorts in the PE (**A**) or AD (**B**) cohort data. All results shown are statistically significant 6 M vs. 0 M (paired *p*_adj_ < 0.05).

Abundance of only 15 species was concurrently altered significatively in the PE and AD cohorts after their respective interventions (Figure 3A and Figure 3B, respectively). The five upregulated lipids in both PE and AD cohorts were phosphatidylinositols, sphingomyelins, and cholesteryl esters, while the eight downregulated lipids in both PE and AD cohorts were diacylglycerols, glycerophosphoglycerols, glycerophosphoethanolamines, and phosphatidylcholines. Most matching species were regulated in the same direction except for two phosphatidylinositols: PI(O-36:2) and PI(O-34:0).

## 5. Discussion

Alterations in the metabolism of lipid species have been associated with obesity and other pathologies [29,30,31,32]. For instance, the dysregulation of the crosstalk between sphingolipids and glycerophospholipids contributes to lipotoxicity-induced metabolic stress [20]. Since the lipidome mainly represents the intermediate and end products of lipid metabolism, lipidomics can be a useful tool to detect physiological dysfunction and earlier stages of changes in metabolism than most clinically used markers [21]. Despite the extensive research concerning adults, lipidomic profiling of children with obesity has been overlooked and often extracted from transversal studies [18,21,33,34].

In the PE cohort, lifestyle intervention led to significant changes in LDL-c, TNF-α, MCP1, IL6, and 71 lipid species circulating levels. The differentially regulated lipids were mainly downregulated (56 vs. 15). Our data showed that the abundance of PI increased due to biosynthesis activation whereas the abundance of PS decreased. Since a decreased abundance of PI and an increased abundance of PS in children with obesity compared to healthy individuals have been demonstrated [18], the observed alteration in the lipidomic profile of PE (6 M) cohort may be interpreted as a positive metabolic response to lifestyle intervention. Specifically, the decrease of circulating levels of PS O-, PS, DG, and PG in PE (6 M) was associated with a trend of metabolic improvement: decreased BMI, weight, insulinemia, and increased total protein. In contrast to other studies, PC was not the lipid species with more significant clinical associations [21,33,34]. Even though the changes in the lipidomic profile of PE (6 M) cohort were not accompanied by a significant weight loss, we could demonstrate a decrease in the inflammatory status. Indeed, numerous studies in children have shown profound changes in biomarkers related to inflammation, oxidative stress, and insulin resistance, as well as some aspects of lipid metabolism after lifestyle intervention, regardless of absolute weight loss [35,36,37,38]. That is precisely the reason why the identification of subclinical changes of metabolic improvement in children with obesity following lifestyle intervention is critical.

In the AD cohort, the abundance of 193 lipids (118 upregulated vs. 75 downregulated) changed 6 M after surgical intervention. In addition, adiponectin and leptin changed toward normalization of their levels (decrease in leptin, increase in adiponectin). Overall, lipid changes after bariatric surgery were in concordance with previous studies [39], including abundance of PC species, specifically ether-linked PCs, that have been reported to be inversely associated to obesity, oxidative stress, and insulin resistance [39]. On the other hand, abundance of TG/DG decreased, which is coherent with metabolic and adipose triglyceride-storing function improvements after bariatric surgery in adult patients [40]. Moreover, correlation analyses revealed a number of previously unexplored associations. A positive correlation was found between PC and HDL. Since PC is an integral component of the lipoproteins, especially HDL [41], this increase in circulating PCs after bariatric surgery could be a consequence of an increase in HDL particles and, therefore, a more favorable lipidic profile. Finally, a positive correlation was also found between TG and insulin, total protein, and urea, suggesting that the decrease of circulating TGs after bariatric surgery may play a role in overall metabolic status improvement in adult obese patients.

Considering the aforementioned lipidomic changes in the AD cohort after bariatric surgery as a lipidomic signature of lipotoxicity reversal, we focused on elucidating whether any of such changes were coincident in the PE cohort after lifestyle intervention, that is, to better distinguish unique changes elicited by routinely advised weight intervention approaches in pediatric patients. We identified fifteen regulated circulating lipid species coincident between AD and PE cohorts at 6 M. Among these, PI(39:3) and PI(41:6) levels were upregulated. Since low PI levels are associated with obesity-induced inflammation, insulin resistance [18] and non-alcoholic fatty liver disease (NAFLD) [42] in childhood obesity, the increase of PI levels triggered by early lifestyle intervention could be interpreted as a sign of metabolic improvement. Additionally, SM(34:2) levels were upregulated. Even though higher concentrations of SM are related to insulin resistance [43], some specific polyunsaturated sphingomyelins have already been described to increase after bariatric surgery [39,44]. In our study, changes in waist circumference negatively correlated with SM(34:2). ST(22:6) levels were also upregulated. Since lecithin-cholesterol acyltransferase (LCAT) activity has been reported to be inversely associated with obesity [45], it is tempting to speculate that increasing CE’s abundance in both cohorts was related to the restoration of normal LCAT functionalities. On the other hand, PC(43:4), DG (36:6)(42:7)(44:9), PG(O 28:0)(O 30:1)(44:0), and PE(52:4) levels were downregulated. Circulating levels of PC, PE, DG, and TG have been positively correlated to insulin resistance in adults [46], so we posited that the downregulation of DG, PE, and PC in the PE cohort after early lifestyle intervention may have a positive impact on insulin resistance in adulthood. Given that circulating PCs are an essential source of triglycerides in steatosis [44], downregulation of PC triggered by early lifestyle intervention in the PE cohort may also have a positive impact on hepatic steatosis. Finally, PI(O-36:2) and PI(O-34:0) were inversely regulated between AD and PE, demonstrating a heterogeneity in the clinical relevance of individual phospholipid species between different age clinical groups, and deserving further characterization to fully interpret its clinical usefulness.

In this pilot study, we acknowledge some limitations. We studied two different cohorts (PE and AD) following two different interventions (lifestyle and bariatric surgery, respectively). We highlight that these two procedures are the currently recommended optimal choices to treat severe obesity in each cohort, since bariatric surgery is not applicable to children with severe obesity, and lifestyle intervention alone in adults with severe obesity has very modest results. These procedures present, nonetheless, some inherent limitations, namely adherence to the intervention in the PE cohort and concomitant dietary changes in the AD cohort. Despite these potential confounders being controlled by the clinicians in charge in regular follow-up visits, additional monitoring methods such as written self-assessment of nutritional intake and physical activity tracking would contribute to reduce the variability introduced by these potential external factors. Moreover, the lack of a necessary control group of children to eliminate nonspecific changes of lipid species with lifestyle intervention would be necessary in future studies. Additional limitations specifically related to the AD cohort, such as ageing, may explain the mismatch between adult and pediatric lipidomic response to weight-loss intervention. Moreover, two different bariatric surgical techniques were included, but, since the anthropometric and metabolic outcomes were not statistically significant, lipidomic changes of both surgical groups could be considered as a whole. For the PE cohort, we acknowledge the limitation of the sample size being small, given that to recruit pediatric patients and obtain consecutive serum samples is restrictive in clinical practice. Finally, in this study we only considered height, weight, waist circumference, and BMI as body composition markers. Inclusion of triceps and subscapular skinfolds, hip circumference, or dual-energy X-ray absorptiometry in further studies would help to better understand the relationship between the changes in pediatric body composition after lifestyle intervention and its association to consequential lipidomic changes. In this pilot study, the levels of some inflammation and oxidative stress biomarkers were measured, while some important free fatty acids including *n*-3 and *n*-6 are lacking. We want to remark that these lipids and their potential correlations with clinical parameters before and after interventions could bring a most interesting perspective in future studies. Moreover, the determination of circulating free fatty acids (FFA) is another limitation of this study, since excess of serum FFA levels is a sign of peripheral insulin resistance [9,10], and therefore could be favorably reduced upon weight loss interventions.

In conclusion, our results indicate that children undergoing lifestyle intervention showed changes in a specific set of lipid species, some of which were coincidental and others differential in adults undergoing bariatric surgery, suggesting that the obtained data constitute a potential pediatric lipidomic signature after interventions aimed at weight loss. Moreover, the baseline results indicate per se a subset of potential biomarkers of obesity at early stages of life. Since no direct clinical value is derived from this data, further studies will be necessary to decipher the potential role of each of the specific lipid species identified in developing obesity-related lipotoxicity and metabolic disorders and potential clinical applications as subclinical biomarkers of such disorders or their improvement after therapeutic interventions, even as molecular targets to counter pediatric and adult obesity.

## Figures and Tables

**Table 1:** Comparison of anthropometrics, demographics, and clinical data between pediatric and adult cohorts at 0 M and 6 M. Quantitative variables are expressed as means and standard deviation (normal distribution) or median and range (non-normal distribution) and the qualitative values as percentages. A *p*-value less than 0.05 was considered as significant (indicated in bold font). Chi-square test, Fisher’s exact test, Student’s t test, and Wilcoxon rank sum test were used to analyze the data when appropriate.

	Pediatric (Total *n* = 10)			Adult (Total *n* = 30)		
	*n* (%) [CI]			*n* (%) [CI]		
Male/female sex	7/3 (70/30)			9/21 (33.3/66.7)		
Sleeve gastrectomy	0 (0)			13 (43.3) [29.9; 70.1]		
Roux-en-Y gastric bypass	0 (0)			17 (56.7) [29.9; 70.1]		
	**mean (sd)**			**mean (sd)**		
Age (years)	14 (2.3)			51.2 (9.3)		
	**Pediatric (Total *n* = 10)**		***p*-Value**	**Adult (Total *n* = 30)**		***p*-Value**
	**mean (sd)**	**mean (sd)**		**mean (sd)**	**mean (sd)**	
	T0	T6		T0	T6	
Height (cm)	160 (10)	160 (10)	0.72	170 (10)	170 (10)	
Weight (kg)	86 (21.3)	83.5 (22.1)	0.42	118.4 (23.3)	87.9 (14.8)	**<0.001**
Waist circumference (cm)	111.6 (11)	110 (14)	0.86	133.8 (13.6)	106.5 (12.4)	**<0.001**
BMI (kg/m^2^) range	34 (5.5) (27.5–43.3)	33.1 (6.1) (25.8–42.8)	0.36	43.2 (5.5) (36.5–65)	32.1 (3.4) (25.3–42.1)	**<0.001**
Glucose (mg/dL)	88.1 (5.7)	87.6 (4.8)	1	101.1 (26.7)	88.4 (10.5)	**0.01**
Insulin (mUI/L)	17.6 (7)	16.5 (6)	0.85	8.9 (7.2)	6.7 (2.4)	0.27
HbA1c (mmol/mol)	33.3 (1.3)	33.3 (1.3)	0.75	37 (8.6)	33.5 (3)	**<0.001**
HOMA-IR	3.9 (1.7)	3.5 (1.3)	0.56	2.4 (2.6)	1.5 (0.6)	0.1
Urea (mg/dL)	27.1 (4)	27.6 (6.5)	0.65	29.8 (16.3)	30.9 (9.1)	**0.03**
Creatinine (mg/dL)	0.6 (0.1)	0.7 (0.1)	0.15	0.8 (0.2)	0.7 (0.1)	0.19
AST (IU/L)	24.8 (10.2)	24.5 (7.8)	1	44.5 (18.4)	21.2 (5.5)	**0.008**
ALT (IU/L)	33.1 (30.3)	23.6 (19.6)	0.19	29.4 (16.4)	21.1 (10.3)	**0.006**
GGT (IU/L)	16.8 (4.0)	16.5 (3.1)	0.76	29.3 (28.7)	21.3 (20)	**<0.001**
TG (mg/dL)	119.4 (53.2)	103.3 (33.6)	0.23	129.8 (41)	86.6 (25.6)	**<0.001**
LDL-c (mg/dL)	114.9 (28.6)	105.4 (24.2)	**0.04**	85.9 (31.4)	107.5 (26.6)	**<0.001**
HDL-c (mg/dL)	43.9 (9.7)	45.4 (8.7)	0.39	37.9 (9.7)	49 (18.3)	**<0.001**
Cholesterol (mg/dL)	177.2 (38.6)	171.1 (32.1)	0.44	149 (38.1)	170.1 (33.1)	**0.005**
Total protein (g/L)	73.4 (4.1)	74.8 (4.5)	0.72	65.2 (12.7)	66.8 (3.9)	0.65
FLI	79.2 (16.0)	75.7 (18.7)	0.29	97.0 (2.6)	61.5 (21.1)	**<0.001**

**Table 2:** Significant correlations between changes in clinical variables and differentially regulated lipids in the PE and AD cohorts 6 M after metabolic intervention.

Expression Pattern	Subclass	Lipid Species	Clinical Variable	Correlation Coefficient	*p*-Value	FDR
**PE (6 M-0 M)**						
Upregulated	SM	SM (39:1) [M+H]1+	Chol	0.6848	0.0289	0.1430
		SM (41:0) [M+H]1+	Chol	0.8303	0.0029	0.0410
		SM (39:1) [M+H]1+	LDL	0.7356	0.0153	0.1046
		SM (41:0) [M+H]1+	LDL	0.8389	0.0024	0.0365
		SM (34:2) [M+H]1+	Waist Circ.	−0.8857	0.0188	0.1108
Downregulated	O-PS	PS-O (36:1) [M+Na]1+	BMI	0.6833	0.0424	0.1821
		PS-O (38:2) [M+Na]1+	BMI	0.6778	0.0448	0.1864
		PS-O (38:4) [M+H]1+/PG-O (38:6) [M+NH4]1+	BMI	0.6833	0.0424	0.1821
		PS-O (36:1) [M+Na]1+	Weight	0.7667	0.0159	0.1057
		PS-O (38:2) [M+Na]1+	Weight	0.7448	0.0213	0.1206
		PS-O (38:4) [M+H]1+/PG-O (38:6) [M+NH4]1+	Weight	0.7667	0.0159	0.1057
		PS-O (36:2) [M+Na]1+	Insulin	0.6442	0.0444	0.1854
		PS-O (36:1) [M+Na]1+	Insulin	0.6809	0.0302	0.1452
		PS-O (38:4) [M+H]1+/PG-O (38:6) [M+NH4]1+	Insulin	0.6809	0.0302	0.1452
		PS-O (38:2) [M+Na]1+	Insulin	0.7301	0.0165	0.1082
		PS-O (38:5) [M+H]1+/PG-P (38:6) [M+NH4]1+	Insulin	0.6442	0.0444	0.1854
		PS-O (34:0) [M+Na]1+	Urea	−0.7150	0.0201	0.1158
		PS-O (36:1) [M+Na]1+	Urea	−0.7256	0.0175	0.1108
		PS-O (36:2) [M+Na]1+	Urea	−0.8923	0.0005	0.0131
		PS-O (36:3) [M+H]1+/PG-O (36:5) [M+NH4]1+	Urea	−0.7323	0.0160	0.1057
		PS-O (36:3) [M+Na]1+	Urea	−0.7330	0.0159	0.1057
		PS-O (38:2) [M+Na]1+	Urea	−0.9354	0.0001	0.0030
		PS-O (38:4) [M+H]1+/PG-O (38:6) [M+NH4]1+	Urea	−0.7256	0.0175	0.1108
		PS-O (38:5) [M+H]1+/PG-P (38:6) [M+NH4]1+	Urea	−0.8923	0.0005	0.0131
		PS-O (38:6) [M+H]1+	Urea	−0.7330	0.0159	0.1057
		PS-O (36:3) [M+Na]1+	Creatinine	−0.6507	0.0416	0.1806
		PS-O (38:6) [M+H]1+	Creatinine	−0.6507	0.0416	0.1806
		PS-O (38:5) [M+Na]1+	TG	−0.6868	0.0283	0.1413
		PS-O (38:5) [M+Na]1+	Total Protein	−0.7254	0.0176	0.1108
		PS-O (40:4) [M+Na]1+	Total Protein	−0.9179	0.0002	0.0060
		PS-O (40:6) [M+Na]1+	Total Protein	−0.7720	0.0089	0.0707
		PS-P (42:2) [M+H]1+	Total Protein	−0.9179	0.0002	0.0060
	PS	PS (38:2) [M+Na]1+	BMI	0.7000	0.0358	0.1639
		PS (38:2) [M+Na]1+	Urea	−0.7561	0.0114	0.0868
		PS (38:4) [M+Na]1+	ALT	−0.7241	0.0274	0.1383
		PS (40:7) [M+H]1+/PG (40:9) [M+NH_4_]1+	ALT	−0.7241	0.0274	0.1383
		PS (38:4) [M+Na]1+	Chol	−0.6659	0.0356	0.1637
		PS (40:7) [M+H]1+/PG (40:9) [M+NH_4_]1+	Chol	−0.6659	0.0356	0.1637
		PS (38:4) [M+Na]1+	Creatinine	−0.7393	0.0146	0.1008
		PS (40:7) [M+H]1+/PG (40:9) [M+NH_4_]1+	Creatinine	−0.7393	0.0146	0.1008
		PS (38:4) [M+Na]1+	TG	−0.7847	0.0072	0.0622
		PS (40:7) [M+H]1+/PG (40:9) [M+NH_4_]1+	TG	−0.7847	0.0072	0.0622
	DG	DG (36:6) [M+H-H_2_O]1+	Insulin	0.7128	0.0207	0.1183
		DG (36:6) [M+H-H_2_O]1+	Weight	0.7120	0.0314	0.1503
		DG (36:6) [M+H-H_2_O]1+	Urea	−0.8655	0.0012	0.0250
	PG	PG (44:0) [M+H]1+	BMI	0.6832	0.0425	0.1821
		PG (44:0) [M+H]1+	ALT	−0.7250	0.0271	0.1383
**AD (6 M-0 M)**						
Upregulated	PC	PC (35:3) [M+H]1+/PE (38:3) [M+H]1+/PA (40:4) [M+NH4]1+	Chol	0.4921	0.0057	0.1558
		PC (35:3) [M+H]1+/PE (38:3) [M+H]1+/PA (40:4) [M+NH4]1+	LDL	0.4179	0.0215	0.3750
		PC (35:3) [M+H]1+/PE (38:3) [M+H]1+/PA (40:4) [M+NH4]1+	HDL	0.5692	0.0010	0.0444
		PC (37:2) [M+H]1+/PE (40:2) [M+H]1+/PA (42:3) [M+NH4]1+	HDL	0.3649	0.0474	0.4911
		PC (37:3) [M+H]1+/PE (40:3) [M+H]1+/PA (42:4) [M+NH_4_]1+	HDL	0.4472	0.0132	0.2858
		PC (40:5) [M+H]1+	AST	0.5091	0.0041	0.1205
		PC (40:5) [M+H]1+	ALT	0.5356	0.0023	0.0810
		PC (42:7) [M+H]1+	ALT	0.3622	0.0492	0.4937
		PC (42:8) [M+H]1+	ALT	0.4208	0.0206	0.3748
		PC (42:7) [M+H]1+	Total Protein	−0.4093	0.0247	0.3793
Downregulated	TG	TG (53:2) [M+Na]1+	Insulin	0.3706	0.0438	0.4694
		TG (53:2) [M+Na]1+	TG	0.8158	0.0000	0.0000
		TG (53:2) [M+Na]1+	Total Protein	0.3971	0.0298	0.3994
		TG (53:2) [M+Na]1+	Urea	0.3921	0.0321	0.4096
		TG (54:2) [M+Na]1+	HbA1c	−0.4011	0.0280	0.3857
	PG	PG (44:0) [M+Na]1+	HOMA.IR	0.3814	0.0376	0.4559

## Data Availability

The datasets generated during and/or analysed during the current study are available from the corresponding author on reasonable request.

## Supplementary Materials

The following supporting information can be downloaded at: https://www.mdpi.com/article/10.3390/nu15153341/s1. File S1: General recommendations list for the treatment of obesity in the PE cohort; File S2: Study flowchart; File S3: Selected lipids for the correlation analyses in the PE(6M) vs. PE(0M) and AD(6M) vs. AD(0M); File S4: Summary of the lifestyle behavioral changes in PE cohort after 6M of lifestyle recommendations; File S5: Adipokines and inflammatory biomarkers response to 6-month intervention, and correlations between adipokines, inflammatory and clinical biomarkers; File S6: Heatmap representation of linear regressions between the differences in FLI and those of selected significantly-regulated lipids coincident in the pediatric (PE) and adult (AD) cohorts; File S7: Statistically significant correlations between the FLI and detectable DG before (0M) and after (6M) 6 months of intervention in PE and AD cohorts; File S8: Age- and sex-adjusted multivariant logistic regression analysis between circulating levels of significantly up and downregulated lipid species 6M-0M after lifestyle intervention and successful body mass index reduction in the PE cohort.
