# Supplementary material for: Lipidome Profiling in Childhood Obesity Compared to Adults: A Pilot Study
Source: Nutrients. 2023 Jul 27;15(15):3341. doi: 10.3390/nu15153341 (PMC10421258; doi:10.3390/nu15153341)
Supplement: Supplementary file 1 [file nutrients-15-03341-s001.zip › Soria_Fernandez-LipidomeandChildhoodObesity-SupplementaryFileS1.pdf]

Lipidome profiling in childhood obesity compared to adults: a pilot study

Andrea Soria-Gondek[1]*, Pablo Fernández-García[2]*, Lorena González[3], Marjorie Reyes-Farias[3,4], Marta Murillo[5], Aina Valls[5], Nativitat Real[6], Silvia Pellitero[7], Jordi Tarascó[8], Benjamin Jenkins[9], María Galán[2], Francesc Villarroya[10,11], Albert Koulman[9], Patricia Corrales[2], Antonio Vidal-Puig[12†], Rubén Cereijo[10,11, 13†], David Sánchez-Infantes[2,3,11†]

Contact info:

David Sánchez-Infantes; Department of Health Sciences, Campus Alcorcón, University Rey Juan Carlos (URJC), E-28922 Madrid, Spain. Electronic address: david.sanchezi@urjc.es

Supplementary file S1

GENERAL RECOMMENDATIONS FOR THE TREATMENT OF OBESITY

These recommendations are designed to **change habits and lifestyle**. The aim is to learn **long-term healthy eating habits**. This is **not** a temporary diet.

1. It is important that you eat calm and relaxed, if possible, not alone or watching television.
2. It is better to eat 5 or 6 meals a day of smaller size, than 3 very large ones. The 3 main meals, breakfast, lunch, and dinner are completed with two snacks in between. This way, you avoid getting very hungry to the main meals.
3. Do not skip meals and try to keep a regular schedule.
4. Serve your meal on a plate and leave the leftovers out of your sight. This will avoid second servings.

5. In addition to diet, physical exercise is advised. You can walk, swim, play tennis, football, cycling or similar. Choose the activity that best suits you and you enjoy the most. You must do sports 2 to 3 days a week, for at least 30 minutes, the more the merrier!
6. It is important that you stay active. The time you spend watching TV must be limited. While you are watching TV, do other activities such as puzzles, crafts, painting, or so one. Counting television, video games and computer time, you should not spend more than 2 hours a day.
7. The whole family should eat the same healthy meal to help you from feeling different.
8. Over time, you will prefer some meals rather than others. You must eat what is made at home. Your family will not cook a different meal for you.
9. The whole family (fathers, mothers, siblings, grandparents, uncles) must collaborate in helping you in your new diet. It is a difficult and long task.

DIETARY RECOMMENDATIONS FOR THE TREATMENT OF OBESITY

1. As a child of growing age, he/she must eat a complete and diversified diet. Sweets should be avoided on a regular basis.
2. Control the intake of fat both from food and additives (butter, margarine, oil).
3. Lower sweets and pastries to a minimum. On special occasions, homemade pastry is advisable.
4. Avoid sugary drinks (Coca-Cola, Pepsi, Fanta, Sunny, etc). They should be reserved for special occasions.
5. The child should drink about 1.5L of water daily.
6. Even though natural juices have a high sugar content; you can give your child a natural juice occasionally. The whole fruit is always better than a juice. Brick fruit juices should be avoided.
7. It is advisable to take 2-3 pieces of fruit daily, except coconut and avocado.
8. The fruity smoothies should be made of 1 fruit and 1 skimmed yogurt/1 glass of skimmed milk, better than of several pieces of fruit.
9. It is advisable to take at least 2 servings of vegetables daily, either raw or cooked.
10. It is advisable to take 3 servings a day of dairy. Milk and yogurts should be skimmed. Cheese should be soft, fresh, or low-fat.
11. Bread, cereals and starches must be present throughout the day. At lunch and dinner, the child can have 1 slice of bread (wholemeal or white bread).
12. Regarding breakfast cereals, Corn Flakes are the ones with less sugar. Special K or Fitness are also accepted. Avoid cereals made with chocolate, honey, sugar, fillings, etc.
13. About cookies, the Maria type is the most advisable.

14. It is advisable to eat more fish than meat. White fish (hake, rooster, sole, monkfish) is better than blue fish (salmon, tuna, sardines, horse mackerel).
15. It is better to consume canned fish (tuna, sardines, cockles) without oil.
16. Choose the leanest meats: chicken, turkey, rabbit and horse. Semi-fat meats (pork and beef tenderloin) can be consumed biweekly. The fattiest meats (lamb, duck, other parts of the pork) can be consumed once every 15 days.
17. Ham, turkey, chicken breast or cured loin can be used to make the mid-morning sandwich or snack (maximum 1 sandwich a day).
18. It is not advisable to take more than 2 or 3 eggs per week.
19. The cooking will be mainly iron, oven, steam, boiled, papillote, microwave ... Maximum 2 times a week fried foods are allowed (stews and batters).
20. Olive oil can be used for cooking and dressing, but moderately. You can also use sunflower or corn oil. Avoid putting oil on sandwiches and bread.
21. Avoid high content sugar food such as candies, gummies, fruit in syrup, marmalades, compotes, jams, chocolate, cocoa, pastries and confectionery.
22. Ready-made foods such as fried fish sticks, breaded chicken, dumplings, croquettes, potato chips, lasagna, etc. are not recommended.
23. If the child stays at school for lunchtime, it is advisable to request the "diet" menu. It is convenient to supervise and plan the home menu according to that of the school, to design a varied and balanced diet.

BALANCED EATING PLAN

BREAKFAST: 1 glass of skimmed milk with 1 teaspoon of powdered chocolate or nescafé.
15 g of cereals OR 4 biscuits OR 20 g of bread

SNACK: Small sandwich with
- tomato (without oil)
- 1 slice of ham, turkey, chicken breast, tuna, fresh or soft cheese

LUNCH: Salad and / or vegetables
Pasta, rice, potato or legume

Meat, fish or egg

1 slice of bread

1 piece of fruit

SNACK: 1 skimmed yogurt with cereals OR 1 fruit OR cookies

DINNER: Salad and / or vegetables.

Pasta, rice, potato or legume (small quantity)

Meat, fish or egg.

1 slice of bread.

1 piece of fruit.

* It is recommended that the daily amount of olive oil be 30 mL (3 tablespoons).

* Perform physical exercise at least 3 days a week, for more than 30 minutes.

commendations, based on out-of-control snacking, sugar craving, introduction of healthy nutrients and physical exercise (2 to 3 times a week, at least 30 minutes). Healthy behaviors are highlighted in green while unhealthy behaviors are highlighted in red.
