# Supplementary material for: Lipidome Profiling in Childhood Obesity Compared to Adults: A Pilot Study
Source: Nutrients. 2023 Jul 27;15(15):3341. doi: 10.3390/nu15153341 (PMC10421258; doi:10.3390/nu15153341)
Supplement: Supplementary file 1 [file nutrients-15-03341-s001.zip › Soria_Fernandez-LipidomeandChildhoodObesity-SupplementaryFileS3.pdf]

Lipidome profiling in childhood obesity compared to adults: a pilot study

Andrea Soria-Gondek[1]*, Pablo Fernández-García[2]*, Lorena González[3], Marjorie Reyes-Farias[3,4], Marta Murillo[5], Aina Valls[5], Nativitat Real[6], Silvia Pellitero[7], Jordi Tarascó[8], Benjamin Jenkins[9], María Galán[2], Francesc Villarroya[10,11], Albert Koulman[9], Patricia Corrales[2], Antonio Vidal-Puig[12†], Rubén Cereijo[10,11, 13†], David Sánchez-Infantes[2,3,11†]

Contact info:

David Sánchez-Infantes; Department of Health Sciences, Campus Alcorcón, University Rey Juan Carlos (URJC), E-28922 Madrid, Spain. Electronic address: david.sanchezi@urjc.es

Supplementary file 3

The selected lipids for the correlation analyses in the PE(6M) vs PE(0M) and AD(6M) vs AD(0M) vs AD(6M), sorted by mainclass division and in decreasing order.

**PE**		**AD**	
**Main class**	**Lipids**	**Main class**	**Lipids**
Phosphatidylserines	PS-O (38:5) [M+Na]1+	Glycerophosphocholines	PC (37:3) [M+H]1+ / PE (40:3) [M+H]1+ / PA (42:4) [M+NH4]1+
	PS-O (36:2) [M+Na]1+		PC (42:8) [M+H]1+
	PS-O (38:5) [M+H]1+ / PG-P (38:6) [M+NH4]1+		PC (42:7) [M+H]1+
	PS-O (36:3) [M+Na]1+		PC (37:2) [M+H]1+ / PE (40:2) [M+H]1+ / PA (42:3) [M+NH4]1+
	PS-O (36:3) [M+H]1+ / PG-O (36:5) [M+NH4]1+		PC (40:5) [M+H]1+
	PS-O (38:6) [M+H]1+		PC (35:3) [M+H]1+ / PE (38:3) [M+H]1+ / PA (40:4) [M+NH4]1+
	PS-O (34:0) [M+Na]1+		PC-O (38:5) [M+H]1+
	PS-O (36:1) [M+Na]1+	Triacylglycerols	TG (53:2) [M+Na]1+
	PS-O (38:4) [M+H]1+ / PG-O (38:6) [M+NH4]1+		TG (47:5) [M+NH4]1+
	PS-O (40:4) [M+Na]1+		TG (54:7) [M+Na]1+
	PS-O (38:2) [M+Na]1+		TG (56:6) [M+Na]1+
	PS-O (40:6) [M+Na]1+		TG (54:2) [M+Na]1+
	PS (38:4) [M+Na]1+		TG (58:8) [M+NH4]1+
	PS (40:7) [M+H]1+ / PG (40:9) [M+NH4]1+	Phosphatidylserines	PS (16:0) [M+H]1+
	PS-P (42:2) [M+H]1+		PS (41:1) [M+H]1+ / PG (41:3) [M+NH4]1+
	PS (38:2) [M+Na]1+		PS-O (41:0) [M+H]1+
Sphingomyelins	SM (41:0) [M+H]1+		PS (34:2) [M+Na]1+
	SM (39:1) [M+H]1+	Phosphatidylinositols	PI (40:9) [M+Na]1+
	SM (34:2) [M+H]1+		PI-O (40:2) [M+NH4]1+
Phosphatidylinositols	PI-O (32:0) [M+H]1+		PI (43:0) [M+Na]1+
	PI-O (41:0) [M+H]1+	Diacylglycerols	DG (42:7) [M+H-H2O]1+
Glycerophosphocholines	PC-O-31:0) [M+H]1+ / PE-O (34:0) [M+H]1+		DG (44:9) [M+H-H2O]1+
	PC (43:4) [M+H]1+	Glycerophosphoglycerols	PG (44:0) [M+Na]1+
Glycerophosphoglycerols	PG (44:0) [M+H]1+		PG-O (34:1) [M+NH4]1+
	PG-O (28:0) [M+H]1+	Glycerophosphates	PA (40:0) [M+NH4]1+
Triacylglycerols	TG (61:14) [M+NH4]1+		PA (39:4) [M+Na]1+

	TG (50:7) [M+NH4]1+	Glycerophosphoethanolamines	PE-O (30:1) [M+H]1+
Diacylglycerols	DG (36:6) [M+H-H2O]1+		LPE (18:4) [M+H]1+
	DG (44:9) [M+H-H2O]1+	Cholesterol esters	CE (22:6) [M+NH4]1+
