# Supplementary material for: Lipidome Profiling in Childhood Obesity Compared to Adults: A Pilot Study
Source: Nutrients. 2023 Jul 27;15(15):3341. doi: 10.3390/nu15153341 (PMC10421258; doi:10.3390/nu15153341)
Supplement: Supplementary file 1 [file nutrients-15-03341-s001.zip › Soria_Fernandez-LipidomeandChildhoodObesity-SupplementaryFileS4.pdf]

Lipidome profiling in childhood obesity compared to adults: a pilot study

Andrea Soria-Gondek[1]*, Pablo Fernández-García[2]*, Lorena González[3], Marjorie Reyes-Farias[3,4], Marta Murillo[5], Aina Valls[5], Nativitat Real[6], Silvia Pellitero[7], Jordi Tarascó[8], Benjamin Jenkins[9], María Galán[2], Francesc Villarroya[10,11], Albert Koulman[9], Patricia Corrales[2], Antonio Vidal-Puig[12†], Rubén Cereijo[10,11, 13†], David Sánchez-Infantes[2,3,11†]

Contact info:

David Sánchez-Infantes; Department of Health Sciences, Campus Alcorcón, University Rey Juan Carlos (URJC), E-28922 Madrid, Spain. Electronic address: david.sanchezi@urjc.es

Supplementary file S4

Summary of the lifestyle behavioral changes in PE cohort after 6M of lifestyle recommendations, based on out-of-control snacking, sugar craving, introduction of healthy nutrients and physical exercise (2 to 3 times a week, at least 30 minutes). Healthy behaviors are highlighted in green while unhealthy behaviors are highlighted in red.

			T0				T6			
**ID**	**Gender**	**Age (Age; Months)**	**Out of control snacking**	**Sugar craving**	**Introduction of healthy nutrients**	**Exercise**	**Out of control snacking**	**Sugar craving**	**Introduction of healthy nutrients**	**Exercise**
**1**	Male	11;6	no	no	partial	no	no	no	partial	no
**2**	Male	13	yes	yes	no	yes	yes	yes	no	yes
**3**	Female	14;5	yes	yes	yes	no	no	no	yes	yes
**4**	Male	17;9	no	no	partial	no	no	no	partial	yes
**5**	Female	17;1	yes	yes	partial	no	no	no	partial	no
**6**	Male	15;1	no	yes	no	no	yes	no	yes	yes
**7**	Male	13;9	no	no	no	no	no	no	partial	no
**8**	Male	10;2	yes	no	partial	no	no	no	partial	yes
**9**	Female	14;1	yes	yes	no	no	no	no	yes	yes
**10**	Male	13;6	yes	yes	yes	no	yes	yes	no	yes
