# Supplementary material for: Lipidome Profiling in Childhood Obesity Compared to Adults: A Pilot Study
Source: Nutrients. 2023 Jul 27;15(15):3341. doi: 10.3390/nu15153341 (PMC10421258; doi:10.3390/nu15153341)
Supplement: Supplementary file 1 [file nutrients-15-03341-s001.zip › Soria_Fernandez-LipidomeandChildhoodObesity-SupplementaryFileS5.pdf]

Lipidome profiling in childhood obesity compared to adults: a pilot study

Andrea Soria-Gondek[1*], Pablo Fernández-García[2*], Lorena González[3], Marjorie Reyes-Farias[3,4], Marta Murillo[5], Aina Valls[5], Nativitat Real[6], Silvia Pellitero[7], Jordi Tarascó[8], Benjamin Jenkins[9], María Galán[2], Francesc Villarroya[10,11], Albert Koulman[9], Patricia Corrales[2], Antonio Vidal-Puig[12†], Rubén Cereijo[10,11, 13†], David Sánchez-Infantes[2,3,11†]

Contact info:

David Sánchez-Infantes; Department of Health Sciences, Campus Alcorcón, University Rey Juan Carlos (URJC), E-28922 Madrid, Spain. Electronic address: david.sanchezi@urjc.es

Supplementary file S5

a. Adipokines and inflammatory biomarkers response to 6-month intervention in the PE cohort (upper row), the AD cohort (middle row) and the difference (Δ, lower row) between 6M-0M. Data shown are means ± standard error of the mean (SEM). Paired samples Wilcoxon test was used to assess differences between 6M and 0M, while one-sample signed-rank test was used to assess differences from unaltered status (null hypothesis: Δ=0 units) in the lower panels compare TNF-α (tumor necrosis factor alfa), MCP1 (monocyte chemoattractant protein-1), IL-8 (interleukin-8) and IL-6 (interleukin-6). *: $p < 0.05$, **: $p < 0.01$, ***: $p < 0.001$, ****: $p < 0.0001$, 6M vs 0M. #: $p < 0.05$, ##: $p < 0.01$, ###: $p < 0.001$, vs Δ=0.

Correlation between adipokines, inflammatory and clinical biomarkers in the pediatric (b) and adult (c) cohorts. Spearman's correlation was conducted for pre-intervention (0M), post-intervention (6M) and difference 6M-0M (Δ) for all variables. Pearson's correlation coefficient (ρ), effective *N* used for each correlation and *p*-values (sig. bilateral) are shown for each correlation; $p < 0.05$ was considered as the threshold of statistical significance and significant correlations were highlighted in green. BMI (body mass index), HOMA-IR (homeostatic model assessment insulin resistance), HbA1c (glycated hemoglobin), CRP (C reactive protein), TG (triglycerides), CHOL (cholesterol), HDL-c (high-density lipoprotein-cholesterol), LDL-c (low-density lipoprotein-

cholesterol, AST (aspartate aminotransferase), ALT (alanine aminotransferase), GGT (gamma-glutamyl transpeptidase).

a. Adipokines and inflammatory biomarkers response to 6-month intervention in PE and AD cohort.

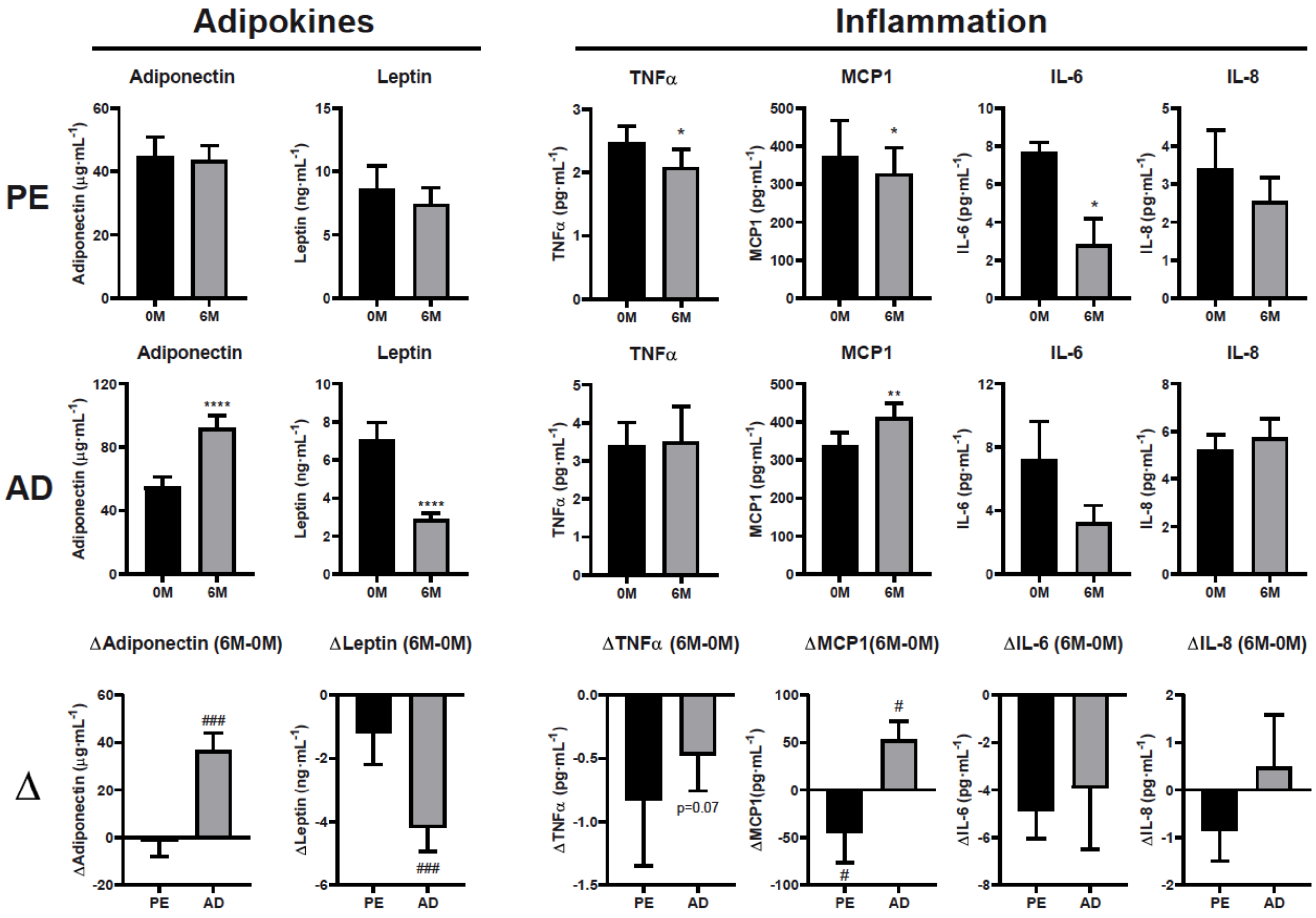

b. Correlation between adipokines, inflammatory and clinical biomarkers. Pediatric cohort.

		0M						6M						Delta					
		Adiponectin	Leptin	TNF-a	MCP1	IL-6	IL-8	Adiponectin	Leptin	TNF-a	MCP1	IL-6	IL-8	Adiponectin	Leptin	TNF-a	MCP1	IL-6	IL-8
**Adiponectin**	Spearman's rho		-0,236	-0,146	0,006	-0,800	0,286		0,152	**0,636**	-0,564	-0,400	0,167		-0,200	-0,115	-0,249		-0,262
	Sig. (bilateral)		0,511	0,687	0,987	0,200	0,493		0,676	**0,048**	0,090	0,600	0,693		0,580	0,751	0,487		0,531
	N		10	10	10	4	8		10	**10**	10	4	8		10	10	10		8
**Leptin**	Spearman's rho	-0,236		0,348	0,152	0,400	0,143	0,152		0,067	-0,273	-0,400	0,643	-0,200		-0,127	0,006		0,524
	Sig. (bilateral)	0,511		0,325	0,676	0,600	0,736	0,676		0,855	0,446	0,600	0,086	0,580		0,726	0,987		0,183
	N	10		10	10	4	8	10		10	10	4	8	10		10	10		8
**TNF-a**	Spearman's rho	-0,146	0,348		-0,037	0,800	-0,491	**0,636**	0,067		-0,006	0,200	-0,167	-0,115	-0,127		0,146		-0,595
	Sig. (bilateral)	0,687	0,325		0,920	0,200	0,217	**0,048**	0,855		0,987	0,800	0,693	0,751	0,726		0,688		0,120
	N	10	10		10	4	8	**10**	10		10	4	8	10	10		10		8
**MCP1**	Spearman's rho	0,006	0,152	-0,037		0,400	0,000	-0,564	-0,273	-0,006		0,800	-0,500	-0,249	0,006	0,146			-0,683
	Sig. (bilateral)	0,987	0,676	0,920		0,600	1,000	0,090	0,446	0,987		0,200	0,207	0,487	0,987	0,688			0,062
	N	10	10	10		4	8	10	10	10		4	8	10	10	10			8
**IL6**	Spearman's rho	-0,800	0,400	0,800	0,400		0,400	-0,400	-0,400	0,200	0,800		0,500						
	Sig. (bilateral)	0,200	0,600	0,200	0,600		0,600	0,600	0,600	0,800	0,200		0,667						
	N	4	4	4	4		4	4	4	4	4		3						
**IL8**	Spearman's rho	0,286	0,143	-0,491	0,000	0,400		0,167	0,643	-0,167	-0,500	0,500		-0,262	0,524	-0,595	-0,683		
	Sig. (bilateral)	0,493	0,736	0,217	1,000	0,600		0,693	0,086	0,693	0,207	0,667		0,531	0,183	0,120	0,062		
	N	8	8	8	8	4		8	8	8	8	3		8	8	8	8		
**Weight**	Spearman's rho	-0,248	0,491	0,384	0,067		-0,238	-0,267	0,533	-0,200	-0,033	0,500	0,500	-0,082	0,119	-0,157	0,243	-0,341	-0,032
	Sig. (bilateral)	0,489	0,150	0,273	0,855		0,570	0,488	0,139	0,606	0,932	0,667	0,207	0,666	0,547	0,444	0,213	0,233	0,887
	N	10	10	10	10		8	9	9	9	9	3	8	30	28	26	28	14	22
**BMI**	Spearman's rho	-0,164	0,612	0,311	0,261	-0,200	0,190	-0,450	0,483	-0,033	0,200	0,500	0,643	0,113	0,165	-0,174	0,004	-0,024	0,041
	Sig. (bilateral)	0,651	0,060	0,382	0,467	0,800	0,651	0,224	0,187	0,932	0,606	0,667	0,086	0,553	0,402	0,395	0,982	0,935	0,855
	N	10	10	10	10	4	8	9	9	9	9	3	8	30	28	26	28	14	22
**Waist Circumference**	Spearman's rho	**-0,775**	0,685	0,482	0,144	0,738	-0,116	0,143	0,600	0,371	0,314		0,429	0,022	-0,054	-0,195	0,211	0,182	0,270
	Sig. (bilateral)	**0,041**	0,090	0,274	0,758	0,262	0,827	0,787	0,208	0,468	0,544		0,397	0,922	0,820	0,438	0,372	0,592	0,294
	N	**7**	7	7	7	4	6	6	6	6	6		6	22	20	18	20	11	17
**Insulin**	Spearman's rho	-0,248	0,442	**0,927**	-0,018		-0,452	-0,418	0,406	-0,115	0,358	0,800	0,429	-0,426	0,134	0,334	0,064	0,500	-0,216
	Sig. (bilateral)	0,489	0,200	**0,000**	0,960		0,260	0,229	0,244	0,751	0,310	0,200	0,289	0,220	0,713	0,345	0,861	0,667	0,608
	N	10	10	**10**	10		8	10	10	10	10	4	8	10	10	10	10	3	8
**Glucose**	Spearman's rho	-0,518	0,055	0,543	-0,104	0,400	**-0,790**	-0,036	-0,188	0,182	0,024	0,200	-0,012	-0,463	0,616	0,140	0,180		0,422
	Sig. (bilateral)	0,125	0,880	0,105	0,776	0,600	**0,020**	0,920	0,602	0,614	0,947	0,800	0,978	0,177	0,058	0,699	0,618		0,298
	N	10	10	10	10	4	**8**	10	10	10	10	4	8	10	10	10	10		8
**HOMAIR**	Spearman's rho	-0,345	0,503	**0,896**	0,079		-0,476	-0,285	0,370	0,176	0,297	0,800	0,500	-0,370	0,297	0,358	0,274	0,500	-0,238
	Sig. (bilateral)	0,328	0,138	**0,000**	0,829		0,233	0,425	0,293	0,627	0,405	0,200	0,207	0,293	0,405	0,310	0,444	0,667	0,570
	N	10	10	**10**	10		8	10	10	10	10	4	8	10	10	10	10	3	8
**HbA1c**	Spearman's rho	0,301	-0,408	-0,060	0,125	0,316	-0,398	-0,325	0,188	-0,375	-0,069	-0,316	0,049	-0,056	0,477	**-0,646**	-0,604		**0,756**
	Sig. (bilateral)	0,398	0,242	0,869	0,730	0,684	0,329	0,359	0,604	0,285	0,850	0,684	0,909	0,877	0,164	**0,044**	0,064		**0,030**
	N	10	10	10	10	4	8	10	10	10	10	4	8	10	10	**10**	10		**8**
**TG**	Spearman's rho	0,049	**0,644**	**0,771**	0,061	0,200	-0,310	0,492	0,620	**0,657**	-0,176	-0,400	0,286	0,042	0,127	**0,794**	-0,079	0,500	-0,381
	Sig. (bilateral)	0,894	**0,044**	**0,009**	0,868	0,800	0,456	0,148	0,056	**0,039**	0,626	0,600	0,493	0,907	0,726	**0,006**	0,828	0,667	0,352
	N	10	**10**	**10**	10	4	8	10	10	**10**	10	4	8	10	10	**10**	10	3	8
**Chol**	Spearman's rho	0,237	0,188	0,187	0,347	-0,400	-0,500	0,164	0,091	0,539	0,406	-0,400	-0,571						
	Sig. (bilateral)	0,510	0,602	0,606	0,327	0,600	0,207	0,651	0,803	0,108	0,244	0,600	0,139						
	N	10	10	10	10	4	8	10	10	10	10	4	8						
**HDL**	Spearman's rho	-0,055	-0,006	-0,110	0,321	-0,400	-0,238	0,055	-0,091	0,224	0,164	-0,400	**-0,857**	**0,760**	-0,109	-0,231	0,012		-0,452
	Sig. (bilateral)	0,881	0,987	0,763	0,365	0,600	0,570	0,881	0,803	0,533	0,651	0,600	**0,007**	**0,011**	0,763	0,521	0,973		0,260
	N	10	10	10	10	4	8	10	10	10	10	4	**8**	**10**	10	10	10		8
**LDL**	Spearman's rho	0,049	0,158	0,006	0,584	0,200	-0,143	0,109	-0,049	0,450	0,365	0,200	-0,476	0,359	-0,018	**-0,723**	-0,256	0,500	0,405
	Sig. (bilateral)	0,894	0,663	0,987	0,077	0,800	0,736	0,763	0,894	0,192	0,300	0,800	0,233	0,309	0,960	**0,018**	0,475	0,667	0,320
	N	10	10	10	10	4	8	10	10	10	10	4	8	10	10	**10**	10	3	8
**Urea**	Spearman's rho	0,213	0,055	0,303	0,395	0,800	0,144	0,328	-0,091	0,225	-0,219	-1,000	-0,611						
	Sig. (bilateral)	0,555	0,881	0,395	0,258	0,200	0,734	0,354	0,802	0,532	0,544		0,108						
	N	10	10	10	10	4	8	10	10	10	10	4	8						
**Creatinine**	Spearman's rho	-0,055	0,127	-0,030	0,358	0,200	-0,452	-0,024	0,036	0,043	0,304	-0,800	-0,479						
	Sig. (bilateral)	0,881	0,726	0,933	0,310	0,800	0,260	0,947	0,920	0,907	0,393	0,200	0,230						
	N	10	10	10	10	4	8	10	10	10	10	4	8						
**AST**	Spearman's rho	0,192	-0,201	0,165	0,351		**-0,786**												
	Sig. (bilateral)	0,620	0,604	0,672	0,354		**0,036**												
	N	9	9	9	9		**7**												
**ALT**	Spearman's rho	-0,164	-0,316	0,217	0,109	0,105	**-0,802**	0,059	0,151	0,050	0,286	-0,949	-0,703						
	Sig. (bilateral)	0,650	0,374	0,547	0,763	0,895	**0,017**	0,881	0,698	0,897	0,456	0,051	0,078						
	N	10	10	10	10	4	**8**	9	9	9	9	4	7						

c. Correlation between adipokines, inflammatory and clinical biomarkers. Adult cohort.

		0M						6M						Delta					
		Adiponectin	Leptin	TNF-a	MCP1	IL-6	IL-8	Adiponectin	Leptin	TNF-a	MCP1	IL-6	IL-8	Adiponectin	Leptin	TNF-a	MCP1	IL-6	IL-8
Adiponectin	Spearman's rho		0,212	0,203	0,239	0,586	0,172		0,008	0,420	0,365	0,049	0,549		-0,006	-0,226	0,048	0,055	0,296
	Sig. (bilateral)		0,279	0,319	0,220	0,008	0,412		0,967	0,033	0,056	0,848	0,008		0,976	0,268	0,809	0,852	0,180
	N		28	26	28	19	25		28	26	28	18	22		28	26	28	14	22
Leptin	Spearman's rho	0,212		0,171	0,274	0,134	0,067	0,008		-0,129	0,048	-0,101	0,082	-0,006		0,014	0,032	0,103	-0,181
	Sig. (bilateral)	0,279		0,403	0,159	0,584	0,751	0,967		0,531	0,810	0,690	0,717	0,976		0,946	0,873	0,725	0,420
	N	28		26	28	19	25	28		26	28	18	22	28		26	28	14	22
TNF-a	Spearman's rho	0,203	0,171		0,796	0,371	0,238	0,420	-0,129		0,554	0,214	0,372	-0,226	0,014		0,178	0,191	-0,238
	Sig. (bilateral)	0,319	0,403		0,000	0,130	0,274	0,033	0,531		0,003	0,394	0,097	0,268	0,946		0,385	0,513	0,311
	N	26	26		26	18	23	26	26		26	18	21	26	26		26	14	20
MCP1	Spearman's rho	0,239	0,274	0,796		0,306	0,248	0,365	0,048	0,554		-0,225	0,332	0,048	0,032	0,178		-0,011	0,495
	Sig. (bilateral)	0,220	0,159	0,000		0,203	0,233	0,056	0,810	0,003		0,369	0,131	0,809	0,873	0,385		0,970	0,019
	N	28	28	26		19	25	28	28	26		18	22	28	28	26		14	22
IL6	Spearman's rho	0,451	0,134	0,371	0,306		0,579	0,049	-0,101	0,214	-0,225		0,473	0,055	0,103	0,191	-0,011		0,287
	Sig. (bilateral)	0,053	0,584	0,130	0,203		0,015	0,848	0,690	0,394	0,369		0,103	0,852	0,725	0,513	0,970		0,366
	N	19	19	18	19		17	18	18	18	18		13	14	14	14	14		12
IL8	Spearman's rho	0,172	0,067	0,238	0,248	0,395		0,549	0,082	0,372	0,332	0,473		0,296	-0,181	-0,238	0,495	0,287	
	Sig. (bilateral)	0,412	0,751	0,274	0,233	0,117		0,008	0,717	0,097	0,131	0,103		0,180	0,420	0,311	0,019	0,366	
	N	25	25	23	25	17		22	22	21	22	13		22	22	20	22	12	
Height	Spearman's rho	-0,233	-0,022	0,387	0,439	0,082	0,256	-0,281	-0,333	0,181	0,160	0,382	-0,128						
	Sig. (bilateral)	0,215	0,911	0,051	0,020	0,739	0,216	0,132	0,084	0,376	0,415	0,118	0,571						
	N	30	28	26	28	19	25	30	28	26	28	18	22						
Weight	Spearman's rho	-0,077	0,292	0,388	0,468	0,375	0,204	-0,043	0,470	-0,031	0,078	0,105	-0,188	-0,082	0,119	-0,157	0,243	-0,341	-0,032
	Sig. (bilateral)	0,688	0,132	0,050	0,012	0,114	0,327	0,820	0,012	0,879	0,692	0,677	0,402	0,666	0,547	0,444	0,213	0,233	0,887
	N	30	28	26	28	19	25	30	28	26	28	18	22	30	28	26	28	14	22
BMI	Spearman's rho	0,072	0,385	0,179	0,189	0,203	-0,127	-0,420	0,043	-0,221	-0,298	0,068	-0,472	0,113	0,165	-0,174	0,004	-0,024	0,041
	Sig. (bilateral)	0,706	0,043	0,382	0,334	0,405	0,545	0,021	0,829	0,278	0,124	0,788	0,027	0,553	0,402	0,395	0,982	0,935	0,855
	N	30	28	26	28	19	25	30	28	26	28	18	22	30	28	26	28	14	22
Waist Circumference	Spearman's rho	-0,204	0,252	0,477	0,600	-0,125	0,133	-0,450	0,015	-0,323	-0,335	0,197	-0,405	0,022	-0,054	-0,195	0,211	0,182	0,270
	Sig. (bilateral)	0,328	0,246	0,029	0,002	0,644	0,565	0,013	0,941	0,108	0,081	0,433	0,062	0,922	0,820	0,438	0,372	0,592	0,294
	N	25	23	21	23	16	21	30	28	26	28	18	22	22	20	18	20	11	17
Insulin	Spearman's rho	-0,202	-0,191	-0,012	-0,073	-0,091	-0,265	-0,034	-0,001	-0,315	-0,112	-0,320	0,147	0,119	-0,035	0,062	-0,127	-0,271	-0,425
	Sig. (bilateral)	0,284	0,332	0,954	0,713	0,710	0,200	0,859	0,997	0,117	0,571	0,196	0,514	0,548	0,864	0,768	0,526	0,349	0,125
	N	30	28	26	28	19	25	30	28	26	28	18	22	28	27	25	27	14	22
Glucose	Spearman's rho	-0,141	0,031	0,061	0,095	-0,304	-0,568	0,276	-0,202	-0,046	-0,032	-0,599	-0,218	0,015	0,369	-0,085	-0,243	-0,029	-0,249
	Sig. (bilateral)	0,456	0,874	0,767	0,631	0,205	0,003	0,139	0,303	0,824	0,871	0,009	0,330	0,938	0,058	0,681	0,223	0,922	0,276
	N	30	28	26	28	19	25	30	28	26	28	18	22	28	27	26	27	14	21
HOMAIR	Spearman's rho	-0,204	-0,211	-0,013	-0,042	-0,121	-0,299	0,340	-0,300	0,181	0,155	-0,084	0,224	0,084	-0,073	0,124	-0,106	-0,361	-0,411
	Sig. (bilateral)	0,280	0,280	0,951	0,830	0,621	0,146	0,066	0,120	0,376	0,432	0,740	0,316	0,667	0,713	0,547	0,591	0,205	0,057
	N	30	28	26	28	19	25	30	28	26	28	18	22	29	28	26	28	14	22
HbA1c	Spearman's rho	-0,185	-0,036	0,025	0,003	-0,180	-0,099	-0,523	-0,084	-0,242	-0,276	0,284	-0,405	-0,048	0,079	-0,084	-0,019	0,399	0,037
	Sig. (bilateral)	0,336	0,857	0,905	0,989	0,462	0,638	0,003	0,671	0,234	0,155	0,253	0,061	0,814	0,709	0,703	0,930	0,158	0,873
	N	29	27	25	27	19	25	30	28	26	28	18	22	27	25	23	25	14	21
CRP	Spearman's rho	-0,096	0,376	0,023	0,485	0,054	-0,055	-0,096	0,376	0,023	0,485	0,054	-0,055	0,238	0,100	-0,259	-0,185	0,515	0,215
	Sig. (bilateral)	0,613	0,049	0,913	0,009	0,828	0,794	0,613	0,049	0,913	0,009	0,828	0,794	0,252	0,649	0,258	0,397	0,087	0,362
	N	30	28	26	28	19	25	30	28	26	28	19	25	25	23	21	23	12	20
TG	Spearman's rho	-0,264	-0,111	0,245	0,089	-0,171	0,008	0,331	0,028	0,097	0,447	0,034	0,391	-0,041	-0,175	0,425	0,251	-0,061	-0,019
	Sig. (bilateral)	0,158	0,575	0,228	0,653	0,483	0,968	0,074	0,889	0,638	0,017	0,893	0,072	0,831	0,382	0,034	0,207	0,844	0,935
	N	30	28	26	28	19	25	30	28	26	28	18	22	29	27	25	27	13	21
Chol	Spearman's rho	0,186	0,279	-0,042	-0,041	-0,215	0,038	-0,079	0,148	0,312	0,079	0,340	0,125						
	Sig. (bilateral)	0,325	0,151	0,838	0,835	0,376	0,858	0,678	0,452	0,120	0,691	0,167	0,578						
	N	30	28	26	28	19	25	30	28	26	28	18	22						
HDL	Spearman's rho	0,433	0,150	-0,085	-0,046	0,219	0,098	0,432	-0,029	-0,372	-0,128	0,062	-0,372	0,200	0,264	-0,278	0,013	0,108	-0,038
	Sig. (bilateral)	0,017	0,447	0,680	0,817	0,367	0,641	0,017	0,885	0,061	0,517	0,807	0,088	0,299	0,183	0,179	0,949	0,714	0,871
	N	30	28	26	28	19	25	30	28	26	28	18	22	29	27	25	27	14	21
LDL	Spearman's rho	0,076	0,312	-0,196	-0,261	-0,238	0,002	0,061	0,221	0,177	0,157	0,246	0,125	0,033	0,077	-0,101	0,024	-0,055	0,313
	Sig. (bilateral)	0,691	0,106	0,336	0,180	0,327	0,994	0,781	0,336	0,468	0,496	0,396	0,657	0,863	0,698	0,622	0,904	0,852	0,156
	N	30	28	26	28	19	25	23	21	19	21	14	15	30	28	26	28	14	22
Urea	Spearman's rho	0,065	0,007	0,004	-0,227	-0,215	-0,209	0,530	0,054	0,195	0,232	-0,227	0,427						
	Sig. (bilateral)	0,734	0,972	0,983	0,245	0,378	0,316	0,003	0,785	0,340	0,235	0,364	0,047						
	N	30	28	26	28	19	25	30	28	26	28	18	22						
Creatinine	Spearman's rho	0,007	-0,016	0,272	0,062	0,048	0,412	-0,028	-0,187	0,048	0,055	0,102	-0,328						
	Sig. (bilateral)	0,971	0,934	0,179	0,752	0,844	0,041	0,885	0,340	0,816	0,780	0,686	0,137						
	N	30	28	26	28	19	25	30	28	26	28	18	22						
AST	Spearman's rho	0,095	-0,086	0,700	0,143	-0,100	0,700	0,307	-0,338	-0,007	0,318	-0,444	0,108						
	Sig. (bilateral)	0,823	0,872	0,188	0,787	0,873	0,188	0,099	0,078	0,974	0,099	0,065	0,633						
	N	8	6	5	6	5	5	30	28	26	28	18	22						
ALT	Spearman's rho	-0,237	-0,151	0,195	0,207	-0,063	-0,203	0,130	-0,183	0,364	0,260	0,207	0,239						
	Sig. (bilateral)	0,207	0,444	0,339	0,290	0,797	0,331	0,493	0,351	0,068	0,181	0,411	0,284						
	N	30	28	26	28	19	25	30	28	26	28	18	22						
GGT	Spearman's rho	-0,287	-0,121	0,148	0,084	-0,183	-0,022	0,405	0,064	0,133	0,344	0,131	0,492						
	Sig. (bilateral)	0,123	0,539	0,471	0,670	0,453	0,916	0,027	0,745	0,518	0,073	0,604	0,020						
	N	30	28	26	28	19	25	30	28	26	28	18	22						
