# Supplementary material for: Lipidome Profiling in Childhood Obesity Compared to Adults: A Pilot Study
Source: Nutrients. 2023 Jul 27;15(15):3341. doi: 10.3390/nu15153341 (PMC10421258; doi:10.3390/nu15153341)

Lipidome profiling in childhood obesity compared to adults: a pilot study

Andrea Soria-Gondek[1]*, Pablo Fernández-García[2]*, Lorena González[3], Marjorie Reyes-Farias[3,4], Marta Murillo[5], Aina Valls[5], Nativitat Real[6], Silvia Pellitero[7], Jordi Tarascó[8], Benjamin Jenkins[9], María Galán[2], Francesc Villarroya[10,11], Albert Koulman[9], Patricia Corrales[2], Antonio Vidal-Puig[12†], Rubén Cereijo[10,11, 13†], David Sánchez-Infantes[2,3,11†]

Contact info:

David Sánchez-Infantes; Department of Health Sciences, Campus Alcorcón, University Rey Juan Carlos (URJC), E-28922 Madrid, Spain. Electronic address: david.sanchezi@urjc.es

Supplementary file S6. Heatmap representation of correlation coefficient (*r*) values for linear regressions between the differences in fatty liver index (FLI) and those of selected top significatively regulated lipids coincident in the pediatric (PE) and adult (AD) cohorts (see main Figure 3) 6 months after metabolic intervention regarding the baseline (Δ6M-0M). Blue cells indicate positive ($r > 0$) and red cells indicate negative associations ($r < 0$). *: Statistically significant correlation ($p < 0.05$).

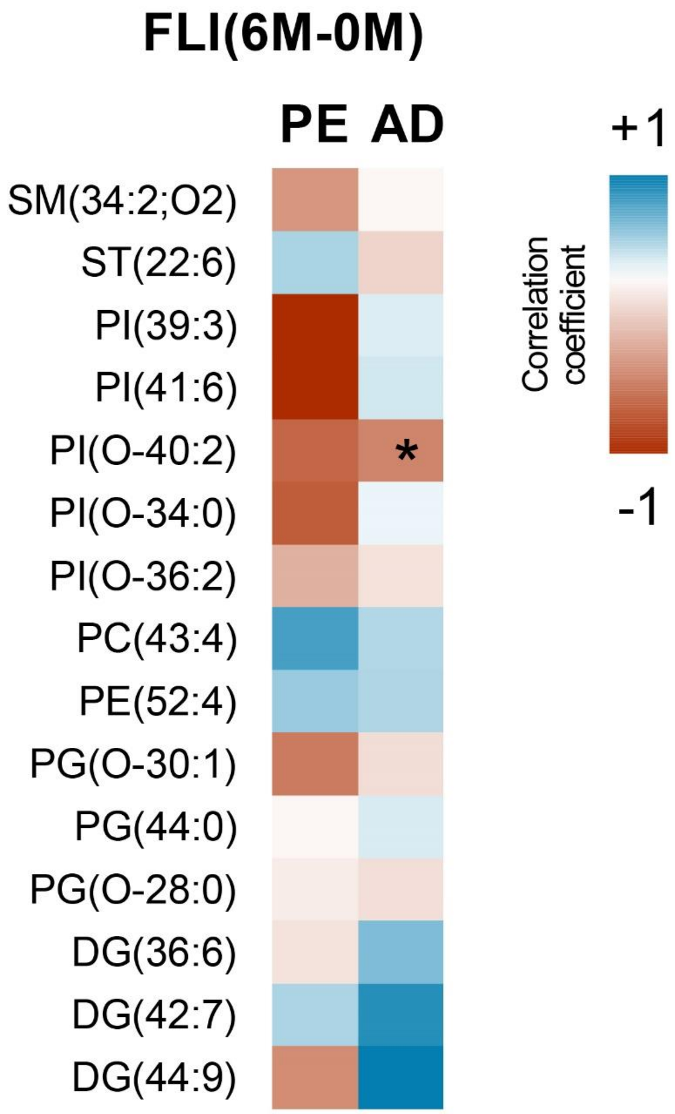

Supplement: Supplementary file 1 [file nutrients-15-03341-s001.zip › Soria_Fernandez-LipidomeandChildhoodObesity-SupplementaryFileS6.pdf]
