# Supplementary material for: Lipidome Profiling in Childhood Obesity Compared to Adults: A Pilot Study
Source: Nutrients. 2023 Jul 27;15(15):3341. doi: 10.3390/nu15153341 (PMC10421258; doi:10.3390/nu15153341)
Supplement: Supplementary file 1 [file nutrients-15-03341-s001.zip › Soria_Fernandez-LipidomeandChildhoodObesity-SupplementaryFileS7.pdf]

Lipidome profiling in childhood obesity compared to adults: a pilot study

Andrea Soria-Gondek[1]*, Pablo Fernández-García[2]*, Lorena González[3], Marjorie Reyes-Farias[3,4], Marta Murillo[5], Aina Valls[5], Nativitat Real[6], Silvia Pellitero[7], Jordi Tarascó[8], Benjamin Jenkins[9], María Galán[2], Francesc Villarroya[10,11], Albert Koulman[9], Patricia Corrales[2], Antonio Vidal-Puig[12†], Rubén Cereijo[10,11, 13†], David Sánchez-Infantes[2,3,11†]

Contact info:

David Sánchez-Infantes; Department of Health Sciences, Campus Alcorcón, University Rey Juan Carlos (URJC), E-28922 Madrid, Spain. Electronic address: david.sanchezi@urjc.es

Supplementary file S7. Statistically significant correlations between the fatty liver index (FLI) and detectable diacylglycerols (DG) before (0M) and after (6M) 6 months of intervention in the pediatric (PE) and adult (AD) cohorts. Statistically significant correlations (Spearman's rho *p*-value < 0.05) are indicated in bold.

	**FLI 0M**		**FLI 6M**	
	**Rho**	***p*-value**	**Rho**	***p*-value**
	**PE**			
DG (36:3); [M+NH4]1+	-0.036	0.939	**0.821**	**0.023**
DG (36:3); [M+H-H2O]1+	0.036	0.939	**0.857**	**0.014**
DG (36:2); [M+NH4]1+	0.179	0.702	**0.857**	**0.014**
DG (36:2); [M+H-H2O]1+	0.071	0.879	**0.857**	**0.014**
DG (35:5); [M+NH4]1+	0.630	0.129	**0.775**	**0.052**
DG (35:3); [M+NH4]1+	-0.450	0.310	**-0.847**	**0.016**
DG (35:2); [M+H-H2O]1+	0.643	0.119	**0.821**	**0.023**
DG (34:3); [M+H-H2O]1+	0.179	0.702	**0.929**	**0.003**
DG (34:2); [M+NH4]1+	0.286	0.535	**0.821**	**0.023**
DG (34:2); [M+H-H2O]1+	0.143	0.760	**0.964**	**<0.001**
DG (33:4); [M+H-H2O]1+	**0.768**	**0.044**	0.556	0.195
DG (33:1); [M+H-H2O]1+	**0.857**	**0.014**	0.643	0.119
DG (32:1); [M+H-H2O]1+	**0.758**	**0.119**	0.643	0.119
DG (30:1); [M+H-H2O]1+	**0.857**	**0.014**	0.286	0.535
DG (30:0); [M+H-H2O]1+	**0.821**	**0.023**	0.179	0.702
	**AD**			
DG (44:11); [M+H-H2O]1+	0.342	0.265	**0.514**	**0.012**
DG (36:4); [M+NH4]1+	**-0.419**	**0.027**	-0.030	0.891
