# Supplementary material for: Lipidome Profiling in Childhood Obesity Compared to Adults: A Pilot Study
Source: Nutrients. 2023 Jul 27;15(15):3341. doi: 10.3390/nu15153341 (PMC10421258; doi:10.3390/nu15153341)
Supplement: Supplementary file 1 [file nutrients-15-03341-s001.zip › Soria_Fernandez-LipidomeandChildhoodObesity-SupplementaryFileS8.pdf]

Supplementary File S8. Age- and sex-adjusted multivariant logistic regression analysis between circulating levels of significantly up and downregulated lipid species 6M-0M after lifestyle intervention and successful body mass index reduction (yes, n = 4; no: n= 6) in the pediatric (PE) cohort. Cox-Snell's R^2 of the multiple regression models and p-value are shown. Associations were considered statistically significant for $p < 0.05$.

**Lipid species**	**Cox-Snell R^2**	***p*-value**
DG 36:6; [M+H-H2O]1+	0.215	0.572
DG 44:9; [M+H-H2O]1+	0.293	0.235
PC 43:4; [M+H]1+	0.143	0.707
PC-O-31:0; [M+H]1+ / PE-O 34:0; [M+H]1+	0.229	0.314
PG 44:0; [M+H]1+	0.190	0.415
PG-O 28:0; [M+H]1+	0.182	0.447
PI-O 32:0; [M+H]1+	0.198	0.477
PI-O 41:0; [M+H]1+	0.151	0.632
PS 38:2; [M+Na]1+	0.131	0.945
PS 38:4; [M+Na]1+	0.299	0.220
PS 40:7; [M+H]1+ / PG 40:9; [M+NH4]1+	0.289	0.199
PS-O 34:0; [M+Na]1+	0.239	0.290
PS-O 36:1; [M+Na]1+	0.242	0.380
PS-O 36:2; [M+Na]1+	0.137	0.782
PS-O 36:3; [M+H]1+ / PG-O 36:5; [M+NH4]1+	0.248	0.282
PS-O 36:3; [M+Na]1+	0.258	0.274
PS-O 38:2; [M+Na]1+	0.590	0.241
PS-O 38:4; [M+H]1+ / PG-O 38:6; [M+NH4]1+	0.242	0.380
PS-O 38:5; [M+H]1+ / PG-P 38:6; [M+NH4]1+	0.137	0.782
PS-O 38:5; [M+Na]1+	0.188	0.430
PS-O 38:6; [M+H]1+	0.258	0.274
PS-O 40:4; [M+Na]1+	0.349	0.285
PS-O 40:6; [M+Na]1+	0.182	0.499
PS-P 42:2; [M+H]1+	0.348	0.285
SM 34:2; [M+H]1+	0.460	0.179
SM 39:1; [M+H]1+	0.154	0.600
SM 41:0; [M+H]1+	0.113	0.878
TG 50:7; [M+NH4]1+	0.446	0.168
TG 61:14; [M+NH4]1+	0.232	0.309
